# Nested sampling for parameter inference in systems biology: application to an exemplar circadian model

**DOI:** 10.1186/1752-0509-7-72

**Published:** 2013-07-30

**Authors:** Stuart Aitken, Ozgur E Akman

**Affiliations:** 1 MRC Human Genetics Unit, IGMM, University of Edinburgh, Edinburgh EH4 2XU, UK; 2 Centre for Systems, Dynamics and Control, College of Engineering, Mathematics & Physical Sciences, University of Exeter, Exeter EX4 4QF, UK

**Keywords:** Model selection, Parameter inference, Nested sampling, Circadian rhythms

## Abstract

**Background:**

Model selection and parameter inference are complex problems that have yet to be fully addressed in systems biology. In contrast with parameter optimisation, parameter inference computes both the parameter means and their standard deviations (or full posterior distributions), thus yielding important information on the extent to which the data and the model topology constrain the inferred parameter values.

**Results:**

We report on the application of nested sampling, a statistical approach to computing the Bayesian evidence *Z*, to the inference of parameters, and the estimation of *log Z* in an established model of circadian rhythms. A ten-fold difference in the coefficient of variation between degradation and transcription parameters is demonstrated. We further show that the uncertainty remaining in the parameter values is reduced by the analysis of increasing numbers of circadian cycles of data, up to 4 cycles, but is unaffected by sampling the data more frequently. Novel algorithms for calculating the likelihood of a model, and a characterisation of the performance of the nested sampling algorithm are also reported. The methods we develop considerably improve the computational efficiency of the likelihood calculation, and of the exploratory step within nested sampling.

**Conclusions:**

We have demonstrated in an exemplar circadian model that the estimates of posterior parameter densities (as summarised by parameter means and standard deviations) are influenced predominately by the length of the time series, becoming more narrowly constrained as the number of circadian cycles considered increases. We have also shown the utility of the coefficient of variation for discriminating between highly-constrained and less-well constrained parameters.

## Background

Choosing rationally between alternative models is one of the most complex and critical problems in systems biology [1]. Given two or more models, and one or more data sets, model selection should identify the model topology and set of kinetic parameters that explains the data best - while simultaneously penalising overly-complex models. A combination of experimental and theoretical arguments can be developed to inform the choice [1]. Calculating the Bayesian evidence (*Z*) is a quantitative approach to answering this question [2,3].

Here, we report on the application of nested sampling - a statistical approach to computing the Bayesian evidence- to the inference of model parameters, and the estimation of *log Z*, in a model of circadian rhythms [4]. An extensive analysis of nested sampling for integration and inference for multivariate Gaussian distributions identifies suitable configurations of nested sampling and associated algorithms in an analytically tractable case. The algorithms employed are generic, simple to configure, or are self-tuning; hence the computational methods can be easily applied in other contexts.

Systems biology models are primarily of interest because they explain data and are capable of making testable predictions [5]. Parameter estimation is a necessary task when a model has been proposed and the parameters that provide the best fit to experimental data must be identified. Modelling and parameter estimation are often interleaved: it may emerge that a model does not have the capability to explain certain features of the data, and the model may be refined as a result. New data may be generated in the lab, and the process of modelling and parameterisation iterates once more. Pokhilko *et al.* describe their revision of the circadian clock in exactly this way [6]. Parameter estimation is central to this process.

Ideally, the representation of the individual reactions that make up a systems model would be based on the known chemistry of the enzymes and substrates involved. However, in practice, there may be uncertainty about the chemical process and its participants. Some species may be assumed to be essentially invariant and excluded from the model, or quantitative values for binding constants may not be known. Often the system is modelled at a level of granularity where multiple chemical steps are represented as a single reaction in order to deal with the complexity of the cell. For example, transcription and degradation are complex processes but are typically each modelled as a single step. As a result, the reactions in a systems model are open to scrutiny, and a justification for modelling decisions is necessary [7].

While justifications in terms of the literature are perfectly valid, and point estimates of the goodness-of-fit to experimental data for some specific combination of parameter values can provide insights and permit model comparison [8], we propose a quantitative measure of evidence derived from the fit of the model to the data. This measure, *log Z*, is the result of an integration (computed as a summation), over the potential parameter values rather than the fit of some exemplar combination of parameters. Evidence calculations can be expected to throw new light on the structure of models.

Nested sampling has been successfully applied in astronomy for model selection and parameter inference [9-11], where cosmological models of up to 42 parameters have been analysed, and techniques for partitioning multi-modal likelihood functions have been developed [12,13]. The properties of nested sampling have been determined for simple models (multivariate Gaussians and mixtures of Gaussians), and the algorithm has been compared to alternative techniques including (annealed) importance sampling [14-16]. Recently, the convergence of nested sampling, its statistical uncertainty and the impact of dimensionality have been addressed theoretically [16-18]. Nested sampling is a valid Monte Carlo technique with convergence rate *O*(*n*^−1/2^) and computational cost *O*(*d*^3^) (where *n* is the size of the population of active points maintained in nested sampling, see below, and *d* is the number of dimensions) [16].

Biological systems models are often simulated or analysed by complex computational procedures. Consequently, the cost of evaluating the likelihood function is often high, and nested sampling must be configured to progress through the prior volume as rapidly as possible without introducing unacceptable errors. We specifically address the problems of sampling the prior (which arises in a complex form in nested sampling [16,19]), defining and exploring the prior volume, and experimentally quantify the uncertainty in the inferred parameter means and standard deviations for a circadian model that is representative of the single feedback loop topology.

In systems biology, an approximate Bayesian computing approach to selecting between alternative models of the JAK-STAT pathway demonstrated strong evidence in favour of one model [1]. Model selection using the Bayes factor (the ratio of the marginal likelihoods of competing models, *P*(*D*|*H*_1_)/*P*(*D*|*H*_2_)) has been shown to be capable of placing an ordering on alternative signal transduction models that is decisive [20]. Bayes factors have been computed for systems models by annealing-melting integration [20] and by population MCMC methods [21,22], techniques which have been made available in the BioBayes package [23]. Nested sampling has been used for DNA motif selection [24], and an application to model selection in systems biology has recently been reported [25] where the MultiNest package developed for cosmology [10] was used to compare signal transduction models of 4, 5 and 6 parameters. We are able to configure nested sampling to run with 25 active points (compared with 1000-10,000 in [25]) thus considerably reducing the number of posterior samples required: For example, on the circadian models we consider here, *log Z* can be estimated to an accuracy of ±0.787 with 25 active points (generating 1200 posterior points and calling the likelihood function 65,500 times), while 1000 active points reduce the variability in *log Z* to ±0.118 but require 35 times the computational effort (generating 42,477 posterior points and calling the likelihood function 2,286,234 times). We also present novel algorithms for the crucial exploratory sub-step of nested sampling, and for the calculation of the transitional likelihood of the systems model.

## Approach

The evidence *Z* (also known as the marginal likelihood) *P*(*D*|*H*_*i*_) is a quantitative measure based on the overall correspondence between the data (*D*) and the model (*H*_*i*_), obtained by integrating the product of the likelihood function and the prior over the space of parameter values [2]. (Recall that the likelihood function *L*(*θ*;*y*) specifies the probability model of the data given the parameters, *P*(*y*|*θ*).) In the inference of the posterior distributions of parameters (1, 2), the evidence plays the role of a normalising constant and need not be evaluated [25]. However, the evidence plays a central role in model comparison and must be computed. The prior assigned to parameters enters into the evidence calculation and can influence the outcome of model comparison through the evidence (or Bayes factor) [26]. Where possible, priors should be selected on the basis of physical considerations [13]: uniform and Jeffreys’ priors have been adopted in cosmology [10,13]; uniform and gamma priors have been employed in biological systems modelling [20-22,25].

(1) $$\begin{array}{lcrr} \text{posterior}=\frac{\text{prior}*\text{likelihood}}{\text{evidence}} & & & \end{array}$$

(2) $$\begin{array}{lcrr} P(\theta |D,H_{i})=\frac{P(\theta |H_{i})P(D|\theta ,H_{i})}{P(D|H_{i})} & & & \end{array}$$

In high dimensions, *log Z* can be computed effectively by a nested sampling strategy that exploits statistical properties of the shrinkage of the prior volume. A set of posterior samples is produced as a by-product of nested sampling, and the first and second moments of parameters can be calculated from these samples. Such an analysis may tell us that certain parameters are very narrowly constrained, while others have a broader distribution, thereby identifying the kinetic parameters for which accurate experimental measurements can validate (or falsify) the model. This computation can also further inform experimental design by quantifying how the parameter estimates depend on the data and experimental protocols used, as well as the extent to which parameters can be constrained by observing particular sets of species. Parameter inference is a challenging problem, and the subject of much on-going research [27].

After determining the number of samples to use in nested sampling, and defining the priors for parameters, we use nested sampling to investigate the posterior distributions of a nine parameter model of circadian rhythms [4]. This analysis is repeated for 1 to 5 cycles of simulated experimental data to explore the impact of time series length on these parameter statistics.

We then explore the problem of model selection for variations of the circadian model with alternative Hill coefficients (*N*=2 − 5), and once more explore the impact of additional data on model selection.

### Nested sampling - an overview

The posterior distribution *P*(*θ*|*D*,*H*_*i*_) of the parameters *θ*, and the evidence *P*(*D*|*H*_*i*_), that is, the support for the data *D* under hypothesis *H*_*i*_, are the two central results of Bayesian inference [3]. Two models *H*_0_ and *H*_1_ can be compared through the ratio of their posterior probabilities (3), a calculation that can be decomposed into the Bayesian evidence (*Z*) and the prior probability of the respective hypotheses which may favour one model over another.

Occam’s razor is implemented as the evidence is proportional to the volume occupied by the posterior relative to the volume occupied by the prior, and hence additional parameters expand the number of dimensions that the evidence must be computed over [11]. The evidence (4) is a scalar quantity that can be viewed as an integral over the elements of mass (d*X*) associated with the prior density *π*(*θ*).

(3) $$\begin{array}{lcrr} \frac{P(H_{1}|D)}{P(H_{0}|D)}=\frac{P(D|H_{1})P(H_{1})}{P(D|H_{0})P(H_{0})}=\frac{Z_{1}P(H_{1})}{Z_{0}P(H_{0})} & & & \end{array}$$

(4) $$\begin{array}{lcrr} Z=\int \;L(\theta )\pi (\theta )\;d\theta =\int \;L(X)\;dX & & & \\ \;dX=\pi (\theta )d\theta & & & \end{array}$$

The prior mass can be accumulated from its elements (d*X*) in any order. Following [3], the cumulant mass of likelihood >*λ* can be defined (5), and this allows the evidence to be written as a one-dimensional integral of the (inverse) likelihood *L*(*X*) over the unit range (taking the enclosed prior mass *X* to be the primary variable) (6).

(5) $$\begin{array}{ll} X(\lambda )= & \int_{L(\theta )>\lambda }\;\pi (\theta )\;d\theta \; \end{array}$$

(6) $$\begin{array}{lll} Z & =\overset{1}{\underset{0}{\int }}\;L(X)\;dX\;\\ & \;L(X(\lambda ))\equiv \lambda \; & \; \end{array}$$

Given a sequence of decreasing values 0<*X*_*m*_<…*X*_2_<*X*_1_<1 where the likelihood *L*_*i*_=*L*(*X*_*i*_) can be evaluated, the evidence can be approximated numerically as a weighted sum (7).

(7) $$\begin{array}{lcrr} Z=\overset{m}{\underset{i=1}{\sum }}L_{i}w_{i} & & & \\ w_{i}=\Delta X_{i} & & & \end{array}$$

**Figure 1:**
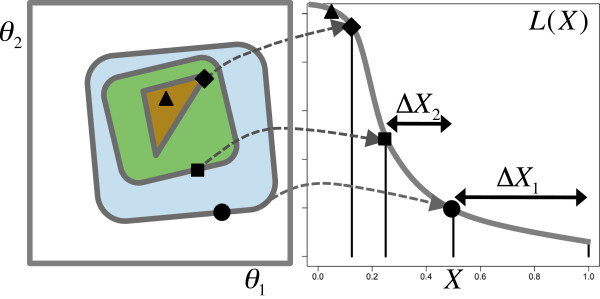
**Nested sampling.** Nested sampling: On each iteration, the worst sample (likelihood = L*) in the set of *n* active points is replaced by a new sample chosen uniformly at random within the likelihood contour defined by L*. For *n*=2, the enclosed prior mass *X* shrinks by a factor of 2 on average as illustrated. The evidence integral is computed from the resulting estimates of *ΔX*_*i*_ and the likelihood of each eliminated sample. Note that the likelihood contours in parameter space (left) are shown schematically and are not assumed to be uni-modal or aligned to the parameter axes.

To obtain the sequence *X*_1_…*X*_*m*_, the nested sampling algorithm maintains a set *S* of *n* active points, each containing a vector of parameter values. On each iteration, the worst point, *x*, is identified (*x*=*argmin*_*i*_*L*(*θ*_*i*_):*i*∈*S*; L* = *L*(*θ*_*x*_)) and replaced by a new point, *z*, drawn uniformly from the prior and subject to the constraint *L(θ*_*z*_*)* >L*. (The worst point contributes a new value *X*_*i*_ to the sequence.) The new point can be found by randomly selecting one of the existing active points (*y*) as a starting point. This procedure shrinks the prior mass geometrically (by the ratio *t*_*i*_=*X*_*i*_/*X*_*i*−1_) according to the beta distribution: $P(t_{i})=\text{Beta}(n,1)=nt_{i}^{n-1}$ for *n* active points [12,14]. The uncertainty in the shrinkage ratio gives rise to an error in *Z* which scales approximately as *n*^−1/2^[2] (the error estimate is refined in [18]). The shrinkage of the prior for a sequence of points from a two-dimensional parameter space is illustrated in Figure 1.

Inferences about the posterior can be obtained from the sequence of *m* discarded points, *P*. Each point is assigned the weight *p*_*i*_=*L*(*θ*_*i*_)*w*_*i*_/*Z*, and the first and second moments of the *j*th parameter in the vector *θ* can be estimated by (8) and (9) respectively.

(8) $$\begin{array}{lcrr} \;\langle \theta_{j}\rangle =\overset{m}{\underset{i=1}{\sum }}p_{i}\theta_{\text{ij}} & & & \end{array}$$

(9) $$\begin{array}{lcrr} \text{var}\;\theta_{j}=\left(\overset{m}{\underset{i=1}{\sum }}p_{i}{\left(\theta_{\text{ij}}\right)}^{2}\right)-{\langle \theta_{j}\rangle }^{2} & & & \end{array}$$

Obtaining new samples *θ*_*z*_ from the truncated prior (that is, uniformly within the specified lower and upper parameter bounds and subject to *L(θ*_*z*_*)* >L*) is a major challenge [19]. Random walk MCMC [2] and rejection sampling within an n-dimensional ellipsoid [11,12] can be used, and these techniques can be coupled to sample multi-modal likelihoods [19]. We explore the use of the stepping out procedure of slice sampling [28] as a simple method of obtaining new points [14]. In the Methods section, we evaluate its use as an exploration method within nested sampling, showing that a single slice sampling step applied in each dimension is sufficient to obtain correct results in up to 30 dimensions using *n*=25 active points. As the prior volume shrinks more rapidly for lower values of *n*, and the number of likelihood evaluations reduces accordingly, it is important to establish this feature of nested sampling’s configuration for practical applications. Adaptively tuning the step size used in stepping out increases computational efficiency significantly and can be done automatically.

### A transitional likelihood function for sparse data

In order for the evidence integral to be computed, a likelihood function *L*(*θ*;*y*) must be defined. For simple stochastic models, such functions may be available in analytic form through solution of the chemical master equation. More generally, systems models will require simulation to obtain a trace of behaviour which can then be compared with the data, or we can approximate the likelihood of the model connecting one observed data point with the next, as we now discuss.

It is shown by [29] that the change *ΔY*(*t*) in a birth-death process *Y*(*t*) is normally-distributed for short time periods *ΔT* over which the rates do not change (and during which many birth and death events take place). Denoting the birth and death rates by *β*(*t*) and *δ*(*t*), respectively, *ΔY*(*t*) is given by (10).

(10) $$\begin{array}{ll} \mathrm{\Delta Y}(t)=Y(t+\mathrm{\Delta T})-Y(t)∼N(\mu ,\sigma ) & \\ \;\mu =(\beta (t)-\delta (t))\mathrm{\Delta T} & \\ \;\sigma =\sqrt{(\beta (t)+\delta (t))\mathrm{\Delta T}} & \end{array}$$

This result motivates the use of stochastic differential equations to model the system dynamics: *β*(*t*) and *δ*(*t*) are derived from the propensities of *Y* in a straightforward manner.

Turning to parameter inference, given a discretely-sampled time series, the likelihood of observing *Y*_1_,…,*Y*_*n*_ is the product $N(Y_{i+1}-Y_{i};\mu ,\sigma )$ for *i*=1,…,*n*−1 where *μ* and *σ* are derived from the birth and death rates as in (10). The likelihood of the model is the product of the likelihood of observing each species. This transitional likelihood function requires the data to be sampled at short time intervals. When the data is sparsely sampled, as is often the case, additional data points can be imputed to bridge the gap between observations. Adopting a Markov Chain Monte Carlo approach, Heron et al. alternate between sampling from the parameter space and sampling from the imputed data space [29]. This strategy is not readily applicable here as the bridge points would need to be included alongside the parameters, thus considerably increasing the dimensionality of the problem, with no obvious way to specialise the treatment of the imputed data.

Noting that the expected change *ΔY*(*t*) is (*β*(*t*)−*δ*(*t*))*ΔT* and that repeated applications of this estimate yield a good predictor of *Y* over time spans many times greater than *ΔT*, we generate the most likely time evolution of the model from the known data vector at *t*_*i*_, impose the condition that the bridge must end at the known data vector at *t*_*i*+1_, and approximate the likelihood of *Y*(*t*_*i*+1_)−*Y*(*t*_*i*_) as the product of the probabilities of the *ΔY*s between the bridge points. This procedure is presented in detail in Methods. The likelihood of each *ΔY* is computed from the cumulative density of the Normal distribution *Φ* (11).

(11) $$\begin{array}{lcrr} L(\mathrm{\Delta Y})=\Phi (\mathrm{\Delta Y}+\varepsilon ;\mu ,\sigma )-\Phi (\mathrm{\Delta Y}-\varepsilon ;\mu ,\sigma ) & & & \end{array}$$

## Results and discussion

After a brief introduction to circadian models, we present the results of parameter inference and model selection obtained using the transitional likelihood function with nested sampling.

### A simple model for circadian rhythms

**Figure 2:**
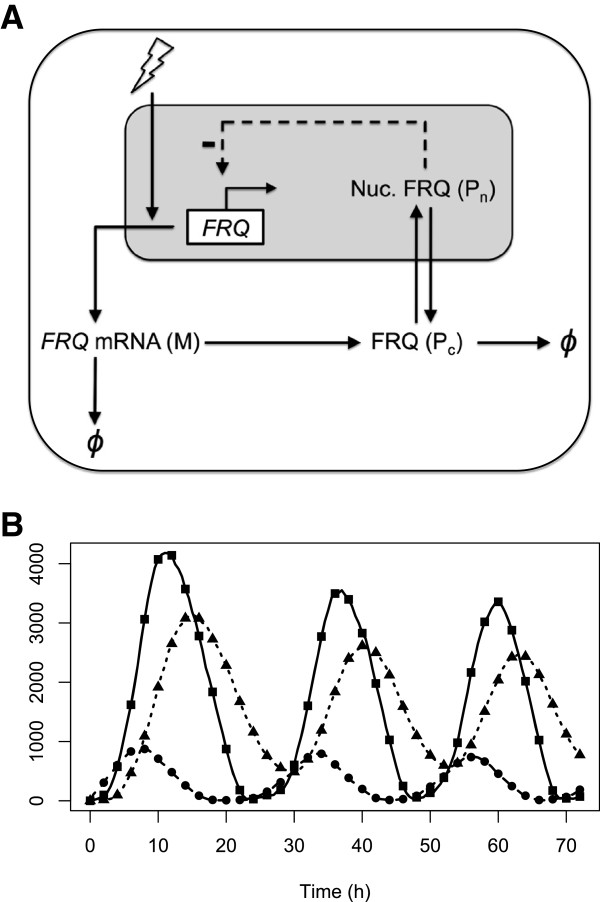
**Model for the circadian clock of *N. crassa*.** Model for the circadian clock of *N. crassa*. **A**. *FRQ* mRNA (M) is translated into protein (P_c_) in the cytoplasm and then transported into the nucleus (P_n_) where it represses the transcription of *FRQ*. Light entrains the model by increasing the transcription rate. **B**. Simulated time series showing sparse samples as symbols: M (circles), P_c_ (squares), and P_n_ (triangles) and the finely-sampled stochastic simulation from which they are selected.

Circadian clocks are gene networks found widely amongst organisms, controlling biological processes ranging from cyanobacterial cell division to human sleep-wake cycles [30]. These networks function by generating endogenous ∼24 hour oscillations in gene expression that can synchronise to the external light-dark cycle. This process, known as entrainment, enables organisms to optimally time biochemical processes relative to dawn and dusk, providing an adaptive advantage [31,32]. The clocks of different organisms appear to have a similar structure based on interlocked sets of negative gene-protein feedback loops augmented by additional positive loops [33]. Computational models of these feedback structures based on ordinary differential equations (ODEs) have become useful tools for quantifying the biochemical mechanisms underlying circadian dynamics [33,34]. Figure 2 shows a minimal ODE model of the clock in the fungus *N. crassa*. This is based on a single negative feedback loop in which the gene *FREQUENCY* (*FRQ*) is repressed by its protein product. *FRQ* transcription is upregulated by light, providing a mechanism for light entrainment [4]. The model comprises 3 differential equations describing the dynamics of *FRQ* mRNA and the cytoplasmic and nuclear forms of FRQ protein:

(12) $$\begin{array}{lcrr} \dot{M} & = & \left(v_{s}+\theta (t)\right)\frac{k_{I}^{N}}{k_{I}^{N}+P_{n}^{N}}-v_{m}\frac{M}{k_{m}+M} & \end{array}$$

(13) $$\begin{array}{lcrr} {\dot{P}}_{c} & = & k_{s}M-v_{d}\frac{P_{c}}{k_{d}+P_{c}}-k_{1}P_{c}+k_{2}P_{n} & \end{array}$$

(14) $$\begin{array}{lcrr} {\dot{P}}_{n} & = & k_{1}P_{c}-k_{2}P_{n} & \end{array}$$

As is common for models of this type, Hill and Michaelis-Menten kinetics are assumed for transcription and degradation respectively, while translation and nuclear transport are modelled as first order reactions. Collectively, the reactions are parameterised by 10 kinetic constants: *v*_*s*_, the maximum *FRQ* transcription rate; *k*_*I*_, the Michaelis constant for *FRQ* repression; *v*_*m*_, the maximum *FRQ* degradation rate; *k*_*m*_, the Michaelis constant for *FRQ* degradation; *k*_*s*_, the FRQ translation rate; *v*_*d*_, the maximum FRQ degradation rate; *k*_*d*_, the Michaelis constant for FRQ degradation; *k*_1_, the rate at which cytoplasmic FRQ enters the nucleus; *k*_2_, the rate at which nuclear FRQ enters the cytoplasm; and *N*, the Hill coefficient. *N* quantifies the binding cooperativity of *FRQ* repression; i.e. the number of sites on the *FRQ* promoter that can be bound by FRQ molecules to prevent transcription. Consequently, it is assumed to be a positive integer [4].

The models and parameter values used in this study are listed in the Additional file 1 (Sections 6 and 7). In equation (12), the forcing term *θ*(*t*) models the effect of light. Setting *θ*=0 simulates constant darkness (DD), yielding free-running oscillations with a period of around 21.5hrs [4]. Entrainment to light-dark (LD) cycles is modelled by switching *θ* between 0 and a maximum value *θ*_*M*_ at lights-on (*t*_*DAWN*_), and then switching *θ* back to 0 at lights-off (*t*_*DUSK*_):

(15) $$\theta \left(t\right)=\left\{\begin{array}{c} \theta_{M}\;\mathrm{if}\;t_{\mathrm{DAWN}}\leq \;\mathrm{mod}\;\left(t,24\right)\leq t_{\mathrm{DUSK}},\\ 0\;\mathrm{otherwise.} \end{array}\right)$$

Birth and death rates for all model species are obtained from the reaction propensities, and are used in the likelihood calculation as described above (10, 11). For example, cytoplasmic protein P_c_ is produced or consumed in the following four reactions (expressed in the reaction syntax of [35]): 

 The birth and death rates (16, 17) for P_c_ follow directly.

(16) $$\begin{array}{lcrr} \beta (t) & = & k_{s}M(t)+k_{2}P_{n}(t) & \end{array}$$

(17) $$\begin{array}{lcrr} \delta (t) & = & v_{d}(P_{c}(t)/(k_{d}+(P_{c}(t)/\Omega )))+k_{1}P_{c}(t) & \end{array}$$

The system size coefficient (*Ω*) is introduced to account for the averaging of stochastic fluctuations that occurs in population-derived data. The likelihood function must be modified as a result.

(18) $$\begin{array}{lcrr} L(\mathrm{\Delta Y})=\frac{\Phi (\mathrm{\Delta Y}+\varepsilon ;\mu ,\sigma )-\Phi (\mathrm{\Delta Y}-\varepsilon ;\mu ,\sigma )}{\Phi (\mu +\varepsilon ;\mu ,\sigma )-\Phi (\mu -\varepsilon ;\mu ,\sigma )} & & & \end{array}$$

When (*β*(*t*)−*δ*(*t*)) approximates the observed *ΔY*, the likelihood in (11) can be maximised by minimising (*β*(*t*)+*δ*(*t*)), as reducing the standard deviation increases the density. Ordinarily this is necessary [36,37]; however, correlations between samples generated from realisations of the circadian model result in the minimisation of (*β*(*t*)+*δ*(*t*)) dominating the fit to the observed *ΔY* to such an extent that the inferred parameter means do not approximate the true values. The normalisation term on the denominator of (18) alters the trade-off between fitting (*β*(*t*)−*δ*(*t*)) to the observed *ΔY* and minimising (*β*(*t*)+*δ*(*t*)), reducing the effect of this bias. *ε* was set to 0.1, and computational explorations showed that the precise value used was not critical to the results obtained.

### Application of nested sampling to the circadian model

Synthetic data ranging from 1 to 5 24 hr circadian cycles was generated using the variant of Gillespie’s stochastic simulation algorithm introduced in [38]. Five time series were generated for each time span in both DD and LD conditions. LD cycles with different daylengths were simulated using equation (15) by setting *θ*_*M*_ equal to 0.8 and varying the size of (*t*_*DUSK*_−*t*_*DAWN*_). All time series were sparsely sampled at 2hr intervals to correspond to typical experimental protocols. Nine of the eleven model parameters were integrated by nested sampling. The Hill coefficient (*N*) and system size (*Ω*=500) were kept constant.

For all rate parameters and constants, a uniform probability density function between positive (non-zero) limits was used as the prior since rates cannot take negative values (without reversing their meaning in the model), and zero values would eliminate the reaction from the model. The circadian model does not contain scale parameters (where relative changes are important) for which the Jeffreys’ prior might be appropriate [2]. The same priors were used for the inference of parameters of the DD model from all realisations of the model, and, similarly, a fixed prior was used for the LD model and in the analysis of alternative values for *N*. As each of the time series generated for each condition is an independent stochastic realisation of the model, the inferred parameter distributions can be expected to include the generating parameters, but this cannot be guaranteed.

**Figure 3:**
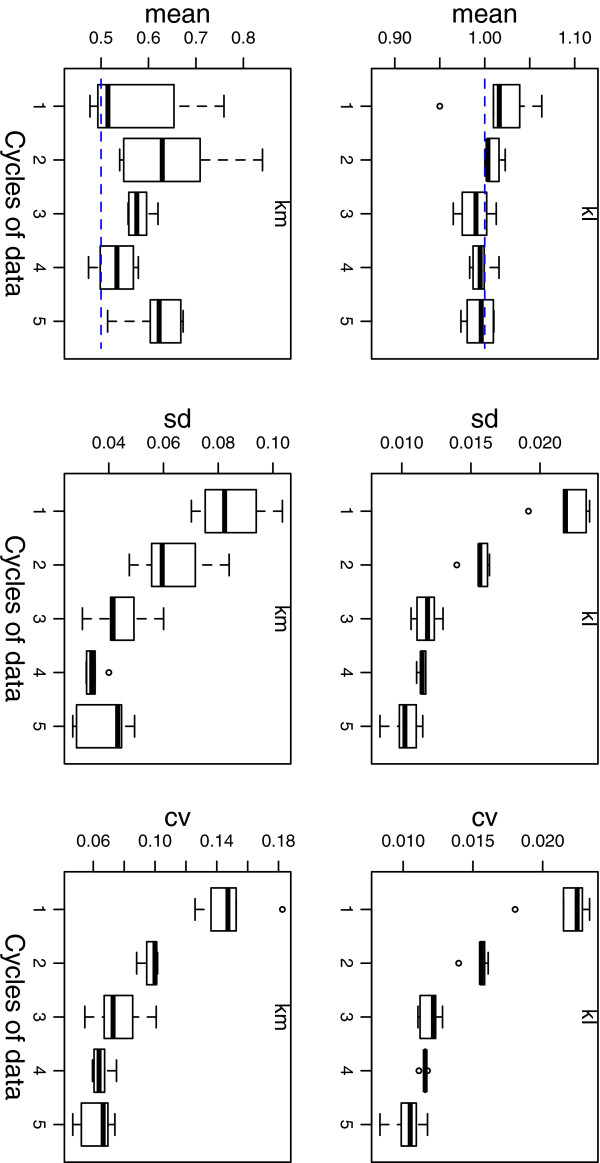
**Parameter inference.** Parameter inference for *k*_*I *_and *k*_*m*_. Estimates of the mean, standard deviation (sd) and coefficient of variation (cv) for model parameters are plotted for 1–5 circadian cycles (24 hrs–120 hrs). Box plots are the result of applying nested sampling to five synthetic data sets generated in simulated DD conditions. The parameter values used to generate the synthetic data were *k*_*I*_=1 and *k*_*m*_=0.5.

The mean, standard deviation (sd) and coefficient of variation (cv = sd/mean) for parameters *k*_*I*_ and *k*_*m*_ are shown in Figure 3 for the free-running (DD) system (see Section 2 of the Additional file 1 for the full results).

Estimates of the mean are broad when inferred from a single cycle of data. These estimates are more precise for 2 or more cycles, with the inferred standard deviation decreasing towards a constant value for 4 or more cycles of data. For *k*_*I*_, the value used to generate the data (1.0) is close to the inferred value for 2 or more cycles of data, whereas for *k*_*m*_ the inferred values are generally higher than the value used in the generating model (0.5). To validate the results, parameter inference was performed on one of the data sets using a standard implementation of MCMC [39] and using nested sampling (see Section 3 of the Additional file 1 and Figure S8). The MCMC simulations confirm that all parameters have a unimodal distribution within the specified priors (see Additional file 1: Figure S8 for parameter distributions), from which it follows that the mean and standard deviations computed by (8) and (9) are meaningful summary statistics. (Posterior samples obtained from multi-modal posteriors can be clustered and analysed separately using the heuristics in MultiNest [10].)

**Figure 4:**
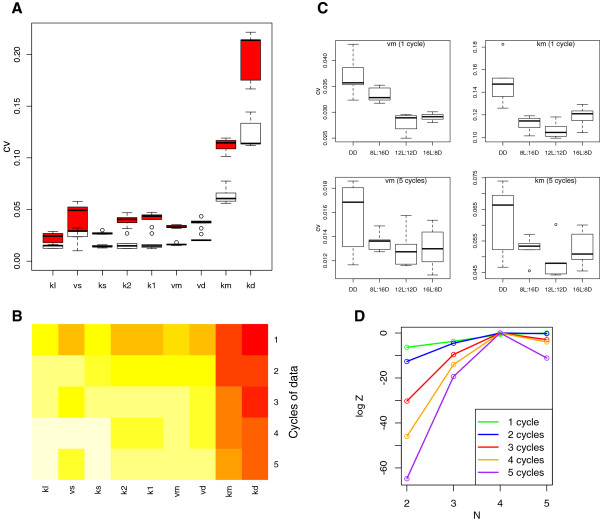
**Coefficients of variation.A**. The cvs for nine model parameters inferred from one cycle (red) and three cycles (white) of DD data. **B**. A heat map showing −*log*(*cv*) for all parameters and cycle lengths on the colour scale white (low cv) through to red (high cv). **C**. The cvs for model parameters *v*_*m*_ and *k*_*m*_ for constant darkness (DD), 8, 12 and 16 hour days, inferred from 1 and from 5 cycles of data. **D**. Calculated *log Z* values for 1–5 cycles of DD data. The maximum *log Z* in each series (cycle length) is set to 0 to facilitate comparison.

Scaling the standard deviation by the mean shows a 10-fold difference between *k*_*I*_ and *k*_*d*_, indicating that the transcription threshold *k*_*I*_ is significantly more tightly constrained (by the model and the data combined) than the protein degradation threshold *k*_*d*_. The cvs for the nine parameters integrated span a wide range as shown in Figure 4A for 1 and 3 circadian cycles, where the parameters are ordered from most to least constrained. The cvs for all parameters and all cycle lengths are plotted as a heat map in Figure 4B. The highly-constrained parameters include *k*_1_ and *k*_2_ – the rates for protein transport to and from the nucleus.

The reduction and convergence of the parameter standard deviations with increasing circadian cycles is not due to the increase in the number of data points (recall that the data is sampled at 2 hr intervals). The total entropy in the data, which is defined as the sum of *plog*(*p*) for all bridge points (*p* is calculated by (18)), increases with the increasing number of data points that result from sampling at uniform intervals over a successively larger number of circadian cycles. Keeping the data set size constant approximately equalises the entropy of the data given the generating model as illustrated in Additional file 1: Figure S9. It can be seen that repeating the parameter inference using a fixed number of 49 samples over 1–3 cycles by varying the sampling interval gives essentially the same results as obtained with varying numbers of data points (Sections 2 and 4 of the Additional file 1 present the results of these two analyses in detail). We conclude that the entropy in the data does not determine the constraints on parameter values for this circadian model.

These results are remarkably consistent given the two sources of variability. Firstly, each of the 25 data series analysed is an independent realisation of the model. Secondly, nested sampling is itself a stochastic procedure. Despite this variation, the analysis is able to quantify the extent to which parameters are constrained, and, further, demonstrates that these constraints vary from parameter to parameter.

Figure 4C (and Additional file 1: Figure S11) show how the parameter distributions are affected by the incorporation of light-dark cycles. The parameter estimation procedure was repeated for the entrained model under the standard protocols of 6L:18D (6 hours in light followed by 18 hours in dark), 12L:12D and 18L:6D. The results indicate that the mRNA degradation parameters *k*_*m*_ and *v*_*m*_ are more tightly constrained under the light-dark cycle protocols, whereas the mRNA transcription rate *v*_*s*_ and P_c_ degradation threshold *k*_*d*_ are less constrained under light-dark cycles, in comparison with DD conditions (see Figure 4C and Additional file 1: Figures S11G and S11I). The remaining parameters show no systematic differences between protocols. All parameters are better constrained by additional cycles of data, consistent with the pattern observed for the free-running system.

Finally, the calculation of *log Z* is proposed as a model selection criterion. Setting the Hill coefficient (*N*) to integer values from 2–5 can be considered to specify different models. As previously, DD data was generated for 1–5 circadian cycles using *N*=4, and nested sampling was applied to these four variations of the circadian model. Figure 4D shows that the Hill coefficient used to generate the data is correctly recovered for 3, 4 and 5 cycles of data. When one or two cycles of data are analysed, the value of *log Z* for the best-fitting model is within one standard deviation of the second best model (that is, we cannot distinguish between *N*=4 and *N*=5 for this particular realisation of the model). For 3 or more cycles, the best model is at least 2.9 log units greater than the second best (*N*=5 in all cases).

Taken together, our results imply that measuring gene expression levels over multiple circadian periods is a more efficient strategy for facilitating robust parameter inference than recording data at high temporal resolutions. For the model considered here, good estimates of parameter means require 2 or more cycles of data, whereas accurate estimates of standard deviations require four or five cycles of data. Reliably determining the Hill coefficient requires at least 3 cycles of data.

The choice of which kinetic constants to measure independently depends on our aims and the specific experimental system of interest. Our results on LD cycles show that this choice may also be partially determined by the experimental protocols used. Here, a true *in vivo* value for the highly-constrained parameter *k*_*s*_ that was substantially different from the inferred value would invalidate the model. However, should we wish to improve the inferred parameter estimates, we should measure the degradation thresholds *k*_*d*_ and *k*_*m*_ as these are more loosely constrained.

## Conclusions

Nested sampling generates a sequence of posterior samples from the parameter space as a by-product of computing the evidence integral. Weighting the parameter values in these samples (points) by the probability of the sample gives estimates of the mean and standard deviation for all model parameters.

The nested sampling algorithm has only one variable, the number of active points *n*. Analysis of Gaussian likelihood functions indicates that 25 active points is sufficient to compute the evidence integral in up to 30 dimensions. The inferred means and standard deviations of parameters are relatively insensitive to the width of the uniform prior we adopt. In contrast, the value of the evidence has some sensitivity to the prior as a narrow prior may omit significant regions of the likelihood integral, whereas the exploration procedure for finding new points may fail to locate all such regions if the prior is too broad. The use of slice sampling permits an automated self-tuning of the exploration procedure that reduces computation significantly. In contrast with Gibbs sampling where 3 or more steps are required [16], we show that a single slice step is sufficient as an exploration procedure.

We have demonstrated in an exemplar circadian model that the estimates of posterior densities (as summarised by parameter means and standard deviations) are influenced predominately by the length of the time series, becoming more narrowly constrained as the number of circadian cycles considered increases. We have also shown the utility of the coefficient of variation for discriminating between highly-constrained and less-well constrained parameters.

In contrast with MCMC approaches, nested sampling has no burn-in period, and does not require complex annealing schedules or output analysis [14]. Nested sampling is therefore well-suited for integration into model analysis software as a robust technique for calculating parameter moments.

## Methods

The R code provided in Additional file 2 implements the nested sampling algorithm and the following refinements:

### Exploration of the prior by slice sampling

The stepping-out procedure of slice sampling [28] is an effective way to explore a uniform prior for a new sample while respecting the constraint *L* >L*, as the step-size can be tuned to increase computational efficiency. Slice sampling is a general-purpose technique: letting *f*(*y*) be a function proportional to the density of the variable *y*, an auxiliary variable *z* is introduced to define a joint distribution over *y* and *z* that is uniform over 0<*z*<*f*(*y*) and 0 elsewhere. After sampling jointly for *y* and *z*, *z* can be ignored and the marginal density *p*(*y*) obtained [28]. Univariate slice sampling takes an initial point as a seed for the generation of a new point that varies from the original in one dimension. After sampling for *z*, the stepping-out algorithm identifies the slice from which *y* is sampled: { *y*:*z*<*f*(*y*)}. This procedure leaves the uniform distribution over the slice invariant [28].

When slice sampling is used within nested sampling, a randomly-selected active point *y* (see main text) is used as the seed. The height of the horizontal slice (the auxiliary variable *z*) is defined by L*. The stepping-out procedure of slice sampling is used to create an interval around *y*, and a new point *y*^′^ is found by selecting a point uniformly at random within the slice { *y*:*L*^∗^<*L*(*y*)} (following the univariate sampling algorithm in [28]). This procedure is applied in each dimension (selected in a random order) to compute a new sample. The use of slice sampling within nested sampling is discussed further in [14].

A novel version of the univariate stepping-out procedure was incorporated into nested sampling. This algorithm included a heuristic to shrink or grow the initial step size as a function of the number of expansion and shrinkage steps made during stepping-out in each dimension in each active point (in addition to the *n* parameter values, each active point object records the slice sample step size to be used in each dimension). The step size associated with the selected dimension is halved when the number of expansion steps is greater than a threshold, or doubled when the number of shrinkage steps is greater than the threshold. In each active point, the slice sample step sizes persist from iteration to iteration of nested sampling, and are copied when a new active point is created from an existing point. The step sizes are tuned for the local region of n-dimensional parameter space. Should the step size be inappropriate - as may occur after the point moves in one of the other dimensions - the efficiency of stepping out may be reduced, but its correctness should not be compromised and the heuristic ensures that a more suitable step size will be used in subsequent iterations. This procedure is effective as new active points take existing samples and their step sizes as the seed, and the step sizes of existing samples are appropriate for the newly-generated sample. This heuristic results in comparable estimates for the evidence (see Figure S1 in the Additional file 1) and reduces the number of likelihood calls to 54–82% of the evaluations required for a fixed step size in tests on 5, 10, 20 and 30 dimensional Gaussian likelihood functions (standard problems for which the solutions are known), as can be seen in Additional file 1: Figure S2.

Next, we determined whether one application of univariate slice sampling produces a new sample sufficiently independent of the seed, or whether a succession of new samples is required. As shown in Figure S3 in the Additional file 1, there is no systematic difference between using one slice step and a series of ten slice steps to calculate *log Z* provided the prior widths are sufficiently large (greater than 6 times the known standard deviation). The integral is underestimated when the prior width is too small, as would be expected. As the prior widths increase, the number of posterior points increases and additional computations of the likelihood are necessary. Computational efficiency is also reduced when the prior widths increase to 40 times the true standard deviation in high dimensions due to the cost of locating the regions of the prior where the likelihood is non-zero.

The combination of nested and slice sampling also correctly estimates the parameter means and standard deviations (see Figure S4 in the Additional file 1). Our investigations into Gaussian likelihoods lead us to conclude that 20–30 active points are sufficient to obtain accurate results in up to 30 dimensions, with the qualification that the width of the uniform prior must be within the identified bounds.

### A stopping criterion for nested sampling

On each iteration of nested sampling, the fraction of the enclosed prior mass (*logWidth*; (19)) and the log of the weight (*logWt*) of the active point with the lowest log likelihood (*ith* posterior point - denoted by the superscript *i*) are calculated (20); and the current estimate of *logZ* updated (21). Finally, the value of log *L*^∗^ is also updated (22). As the nested sampling algorithm progresses through the prior volume, the *logL* of each posterior point is guaranteed to increase on each iteration, and *logWidth* reduces. The weight given to a posterior point initially increases as *logL* increases, then this weight begins to decrease as the reduction in *logWidth* outweights the increase in *logL*[2].

(19) $$\begin{array}{lcrr} \text{logWidth} & = & \text{log}((e^{-(i-1)/n}-e^{-(i+1)/n})/2) & \end{array}$$

(20) $$\begin{array}{lcrr} \text{logW}t^{i} & = & \text{logWidth}+\text{log}L^{i} & \end{array}$$

(21) $$\begin{array}{lcrr} \text{logZ} & = & \mathrm{log.plus}(\text{logZ},\text{logW}t^{i}) & \end{array}$$

(22) $$\begin{array}{lcrr} \text{log}L^{*} & = & \text{log}L^{i} & \end{array}$$

The stopping criterion requires the current *logWt* values to be compared with those of the posterior point identified 50 iterations earlier. At any iteration *i*, the difference in log width is *-50/n* (23). Should the change in *logWt* be primarily due to the reduction in log width, with little or no contribution from increased *logL*, the informative region of the prior must have been explored and nested sampling can terminate. Comparing *logWt* values 50 iterations apart is more robust than comparing values at successive iterations as *logWt* is not guaranteed to change monotonically and often exhibits a noisy characteristic from iteration to iteration.

(23) $$\begin{array}{lcrr} \text{log}((e^{-(i+50-1)/n}-e^{-(i+50+1)/n})/2)= & & & \\ \text{log}((e^{-(i-1)/n}-e^{-(i+1)/n})/2)-50/n & & & \end{array}$$

Nested sampling can be terminated when the difference between log weights assigned to posterior points obtained 50 iterations apart tends to: 50/*n* (*logL*^∗^ must also be significantly greater than *logWt*^*i*^ to prevent early termination). The maximum number of posterior points can also be specified in order to bound the computation. The change in the log likelihood assigned to posterior points obtained 50 iterations apart can also be tested. All runs of nested sampling on the DD model terminate by the test on *logWt*. For example, one run terminates with a change in *logWt*<1.996 (and *logL*^∗^−*logWt*^*i*^=56.5), whereas the change in *logL*^∗^ over those iterations is 0.00396 which, by itself, does not necessarily indicate termination.

### Bridging sparsely-sampled data

The experimental data is assumed to be sampled at intervals that are many times longer than that at which the transitional likelihood can be applied directly (10). Noting that the expected change *ΔY*(*t*) is (*β*(*t*)−*δ*(*t*))*ΔT* over short intervals, and that repeated applications of this estimate yield a good predictor of the deterministic trajectory of *Y* over time spans many times greater than *ΔT*, we generate the most likely time evolution of the model over a sequence of short time intervals starting from the known data vector at *t*_*i*_, then impose the condition that this *bridge* must end at the known data vector at *t*_*i*+1_, and approximate the likelihood of *Y*(*t*_*i*+1_)−*Y*(*t*_*i*_) as the product of the probabilities of the *ΔY*s between the bridge points. The likelihood of each *ΔY* is computed from the cumulative density of the Normal distribution *Φ*.

To illustrate the method, two observed data points are represented by the black crosses at 1h (B1) and 4h (B4) in Additional file 1 : Figure S5A. The bridge points B2, B3 and B end are generated in sequence from B1 by (10). Where the expected value of B end does not correspond to the known value B4, the bridge points are scaled to new values B2 ^′^ and B3 ^′^. The likelihood of *Δ*B2 ^′^ is assessed using the prediction made from B1 (*μ*=*Δ*B2 by construction). The likelihood of *Δ*B3 ^′^ is assessed using the prediction made from B2 ^′^, a scaled point.

A bridge of *l* steps requires *l*−1 bridge points. Let *B* be an array of *l*+1*n*-dimensional points such that *B*[ 1]=*D*[ *t*_*i*_] and *B*[ *l*+1]=*D*[ *t*_*i*+1_]. Bridge points B[2],…,B[l] are generated by (10) from the previous point in the sequence, and the projected end point *B*_*end*_ corresponding to *t*_*i*+1_ is generated from *B*[ *l*]. In each dimension *d*, the error at the bridge end *r*_*d*_=*B*_*end*_[ *d*]/*B*[ *l*+1][ *d*], is used to correct the trajectory by assigning the error to B[2][d],…,B[l][d] proportionally as illustrated in Additional file 1: Figure S5A. Bridge points *B*[ *i*][ *d*] are multiplied by (1+(*i*−1)(*r*_*d*_−1)/*l*) for i=2,…,l. For parameter values yielding birth and death rates that bridge the data perfectly, *r*=1 and the correction has no effect on the bridge. Otherwise, the correction constructs a plausible path between the known end points that is scaled from the expected, but ill-fitting, path originating at *D*[ *t*_*i*_]. Additional file 1: Figure S5B shows a typical result of bridging data sampled at 2 hour intervals with a bridge of length 20 computed using the heuristic described above.

The proposed bridging technique was evaluated by comparison with simulated annealing on the task of computing the log likelihood of a bridge between two data points 〈M_1_, P_c1_, P_n1_〉, 〈M_2_, P_c2_, P_n2_〉 for a specified set of model parameters. This is the elementary problem that must be solved *m*−1 times for *m* sparsely-sampled data points. A simulated annealing algorithm was implemented to find the optimal bridge points by search. A bridge length of 10 was found to be practical (generating a 30 dimensional search problem). The end data point 〈M_2_, P_c2_, P_n2_〉 was moved in all three dimensions to generate a space of likelihood values. The log likelihood values calculated by bridging are compared with those obtained by simulated annealing in Additional file 1: Figure S6. The left panel of Additional file 1: Figure S6 shows the correlation between the likelihood values computed by the alternative methods, and the 95% confidence interval around the mean obtained for 10 repeats of simulated annealing (Pearson correlation 0.79). The right panel of Additonal file 1: Figure S6 shows the relationship between the predictions for larger displacements of the end data point (Pearson correlation 0.93). Simulated annealing took an average of 15s to compute one series of bridge points. The bridge heuristic took 0.0025s to compute the bridge - 6000 times faster than simulated annealing.

## Competing interests

Both authors declare that they have no competing interests.

## Authors’ contributions

SA and OEA performed and designed research and wrote the paper. Both authors read and approved the final manuscript.

## Supplementary Material

Additional file 1 **Supplementary Material.** Contains: supplementary figures; full parameter inference results for simulated DD and LD conditions; comparisons of the nested sampling results with an MCMC-based method; results demonstrating the effect of varying the sampling interval; and details of the models and parameter values used to generate the synthetic data sets. Click here for file

Additional file 2 **R Code for Nested Sampling.** R code for nested sampling, slice sampling and for the transitional likelihood computation can be found in NestedSamplingRCode.tar. Click here for file
