# Supplementary material for: Nested sampling for parameter inference in systems biology: application to an exemplar circadian model
Source: BMC Syst Biol. 2013 Jul 30;7:72. doi: 10.1186/1752-0509-7-72 (PMC3735395; doi:10.1186/1752-0509-7-72)
Supplement: Additional file 1 — Supplementary Material. Contains: supplementary figures; full parameter inference results for simulated DD and LD conditions; comparisons of the nested sampling results with an MCMC-based method; results demonstrating the effect of varying the sampling interval; and details of the models and parameter values used to generate the synthetic data sets. [file 1752-0509-7-72-S1.pdf]

Nested sampling for model selection and parameter inference in systems biology - Supplementary Material

Stuart Aitken[1*] and Ozgur E. Akman[2]
1 School of Informatics and SynthSys, University of Edinburgh
2 Centre for Systems, Dynamics and Control, College of Engineering, Mathematics & Physical Sciences, University of Exeter

Contents

1 Supplementary Figures **2**

2 Parameter inference for the free-running (DD) system **8**

3 Comparison of nested sampling and MCMC **14**

4 Parameter inference for the free-running (DD) system with constant sample size **18**

5 Parameter inference for LD protocols **25**

6 Free-running Circadian Model **35**

7 Entrained Circadian Model **36**

1 Supplementary Figures

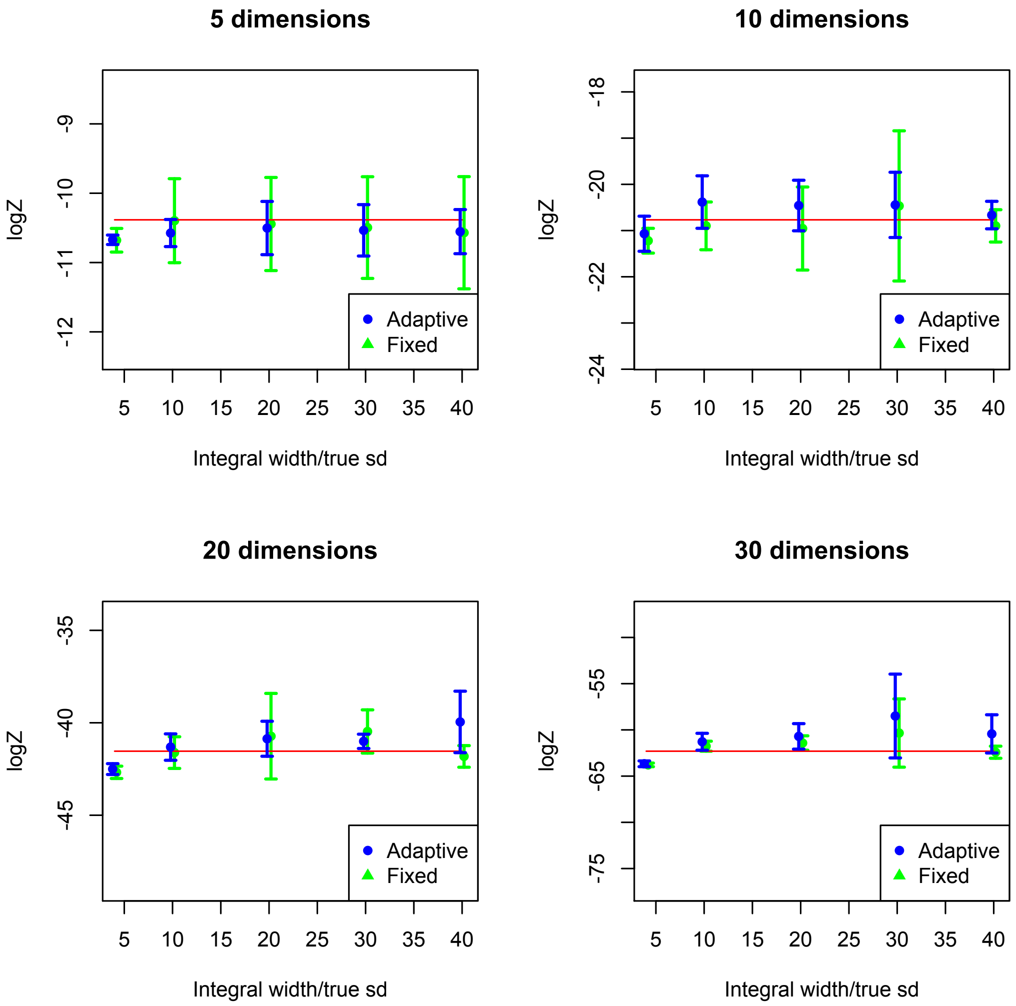

Fig. S1. Calculation of *log Z* for 5, 10, 20 and 30-dimensional isotropic Gaussian likelihood functions ($e^{-\Sigma(X_i-\mu)^2/2\sigma^2}$) over prior widths from 4 to 40 standard deviations (σ=0.05). The mean estimates of *log Z* (error bars indicate 95% confidence intervals) were obtained by nested sampling using 25 active samples (n=25) from 5 runs of the algorithm. The true values of *log Z* are indicated by the red lines (-10.38, -20.77, -41.54 and -62.30 in 5, 10, 20 and 30 dimensions respectively). Nested sampling computes *log Z* in an n-dimensional cube 0..1 from the posterior points obtained. By design, these points are sampled from the region(s) of the prior where the likelihood is non zero. The final estimate of *log Z* is obtained by multiplying the output of nested sampling by the volume of the prior in parameter space $(d_1^{max} - d_1^{min})..*(d_n^{max} - d_n^{min})$ where the likelihood is not zero. When the integral width becomes large (30 times the true standard deviation, or more) in higher dimensions, the proportion of the prior where the likelihood is greater than zero becomes significantly smaller than the volume of parameter space, and therefore we scale this volume by an estimate of the non-zero fraction. This estimate is obtained in an independent analysis by evaluating the likelihood in up to 10^7 random samples from the parameter space.

Results obtained using 10 steps of slice sampling with the step size updated adaptively (blue) do not differ systematically from those obtained with a fixed step size (green).

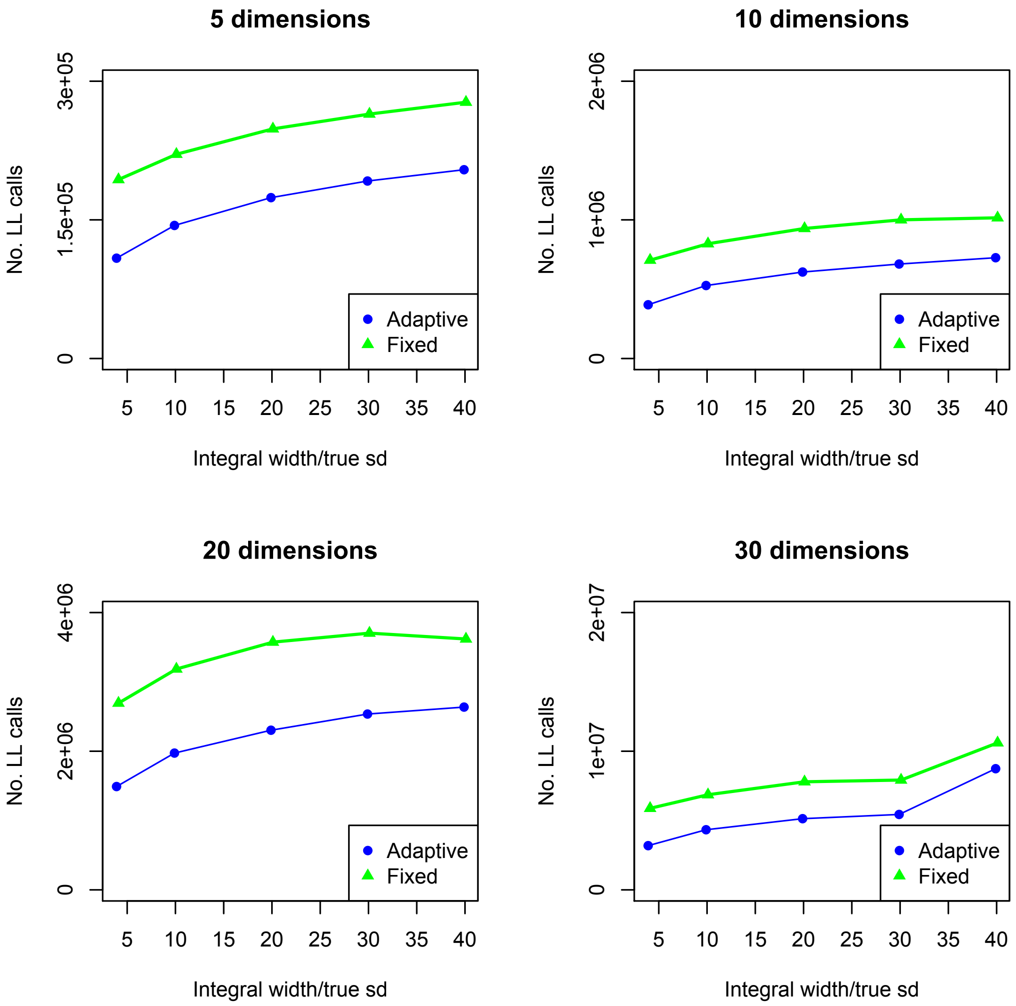

Fig. S2. Computational cost of nested sampling. The average number of log likelihood (LL) calls required using 10 steps of slice sampling with the step size updated adaptively (blue) and with fixed initial step size (green) for the runs of Fig. S1 are shown. The adaptive heuristic for setting the step size reduces the number of likelihood calls to 54-82% of the evaluations required for a fixed step size.

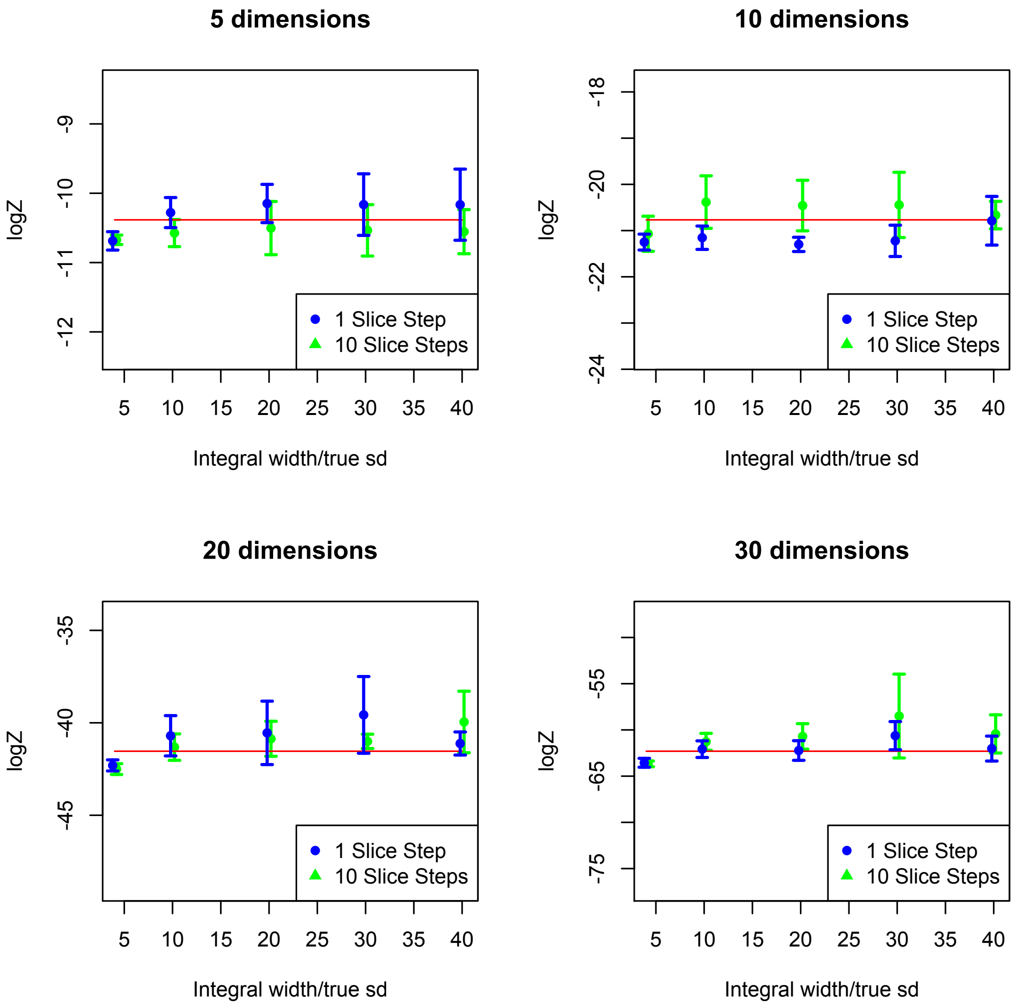

Fig. S3. Calculation of *log Z* for 5, 10, 20 and 30-dimensional Gaussian likelihood functions ($e^{-\Sigma(X_i-\mu)^2/2\sigma^2}$) over prior widths from 4 to 40 standard deviations (σ=0.05). The mean estimates of *log Z* (error bars indicate 95% confidence intervals) were obtained by nested sampling using 25 active samples (n=25) from 5 runs of the algorithm. The true values of *log Z* are indicated by the red lines (-10.38, -20.77, -41.54 and -62.30 in 5, 10, 20 and 30 dimensions respectively). Results obtained using 1 step of slice sampling in the exploration step (blue) do not differ systematically from those obtained using 10 steps of slice sampling (green). The adaptive heuristic is used in both cases.

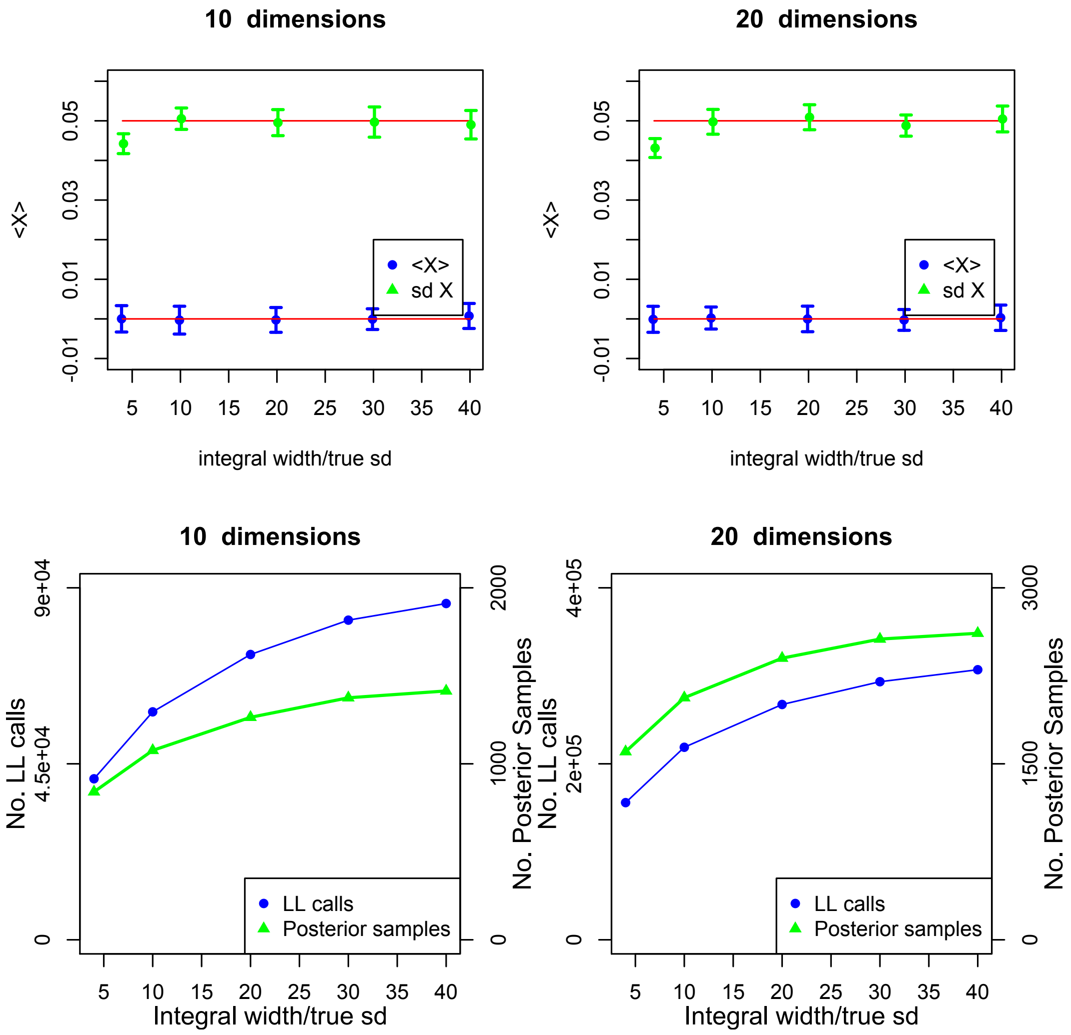

Fig. S4. Inferred parameters and the computational cost of nested sampling. Top left and top right, inferred values of the mean μ (blue) and standard deviation σ (green) of the Gaussian-distributed parameter X: the true values are 0 and 0.05, respectively, in all dimensions as indicated by the red lines. Results were obtained using 1 slice sampling step to integrate over prior widths from 4 to 40 standard deviations in 10 and 20 dimensions. Symbols represent the mean of all estimates obtained in all repeats of nested sampling (for the specified number of dimensions and integral width), error bars denote the standard deviation of these estimates. Bottom left and bottom right, the average number of log likelihood (LL) calls (blue) and average number of posterior samples generated (green) in 10 and 20 dimensions. These data and those of Fig. S3 top right (10 dimensions) and bottom left (20 dimensions) are derived from the same series of 5 repeated runs of nested sampling.

A

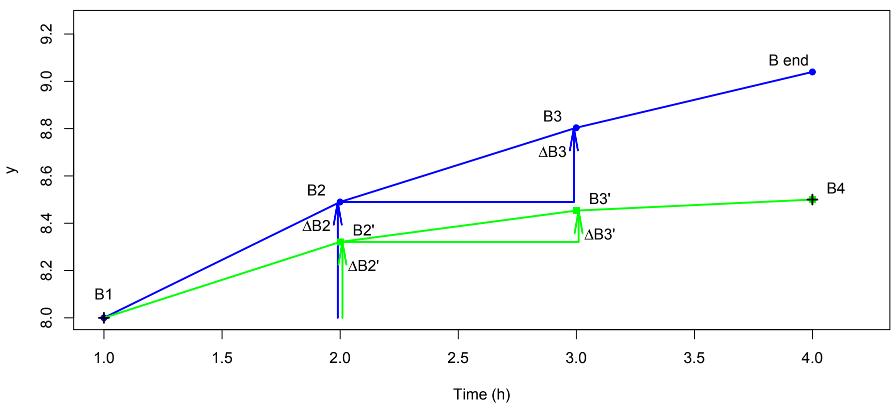

B

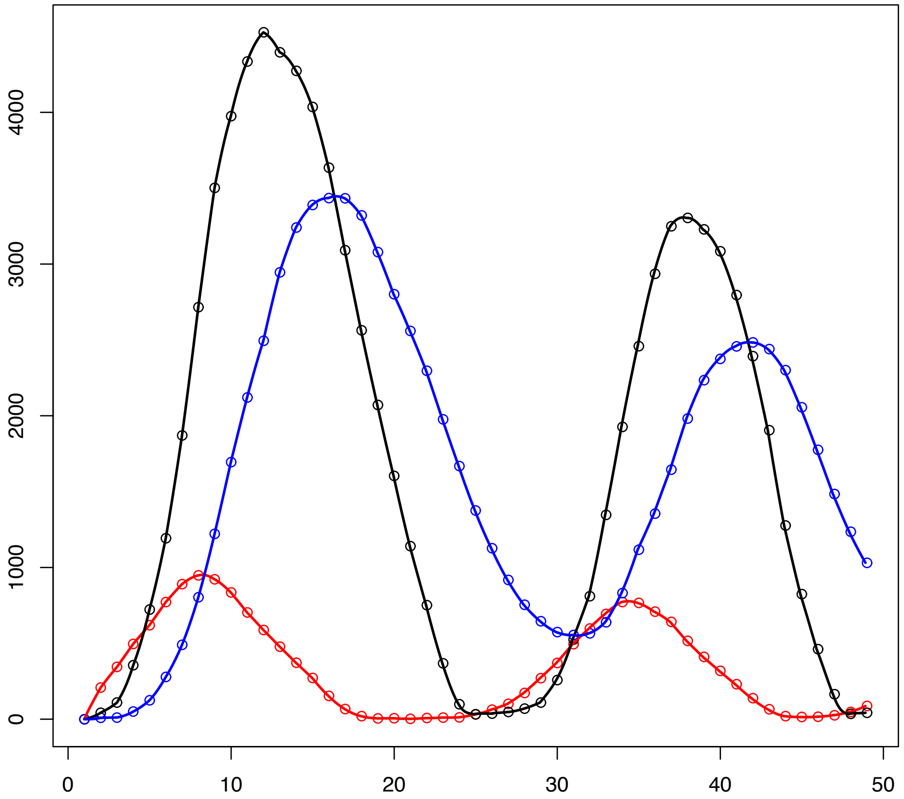

Fig. S5. Bridging sparse data. **A**. Schematic of bridging for a bridge length of three. The observed data is represented by the black crosses at 1h (B1) and 4h (B4). Initially, the bridge points B2, B3 and B end are generated in sequence from B1 by (equation (10)). In this example, the expected value of B end does not correspond to the known value B4, and as a consequence the bridge points are scaled to new values B2′ and B3′. **B**. Sparsely-sampled data (circles) and bridge points (solid lines) computed as in A for M (red), P_c (black) and P_n (blue) over two circadian cycles.

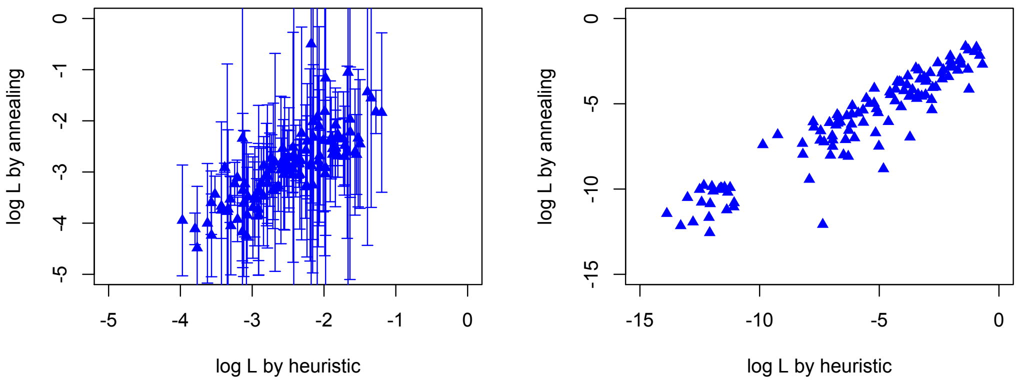

Fig. S6. A comparison of log likelihood values calculated by simulated annealing and by the bridge heuristic. Left panel shows a scatterplot of the log likelihood values for small displacements of the end data point (95% confidence intervals are derived from 10 repeats of simulated annealing). Right panel shows a scatterplot of log likelihood values for larger displacements of the end data point.

2 Parameter inference for the free-running (DD) system

Fig. S7. Parameter inference for the free-running (DD) system over 1-5 circadian cycles of data. All figures show box plots summarising 5 runs of the nested sampling algorithm. Each run used a different stochastic realisation of the circadian model. The same (uniform) prior range for parameters was used in all runs. The results are grouped and plotted in several different ways to ease comparison. The parameter values used to generate each of the synthetic data series are indicated by the blue dashed lines.
A. Mean of all parameters as a function of the number of cycles. Triangles indicate the upper and lower bound used for integration (prior width).
B. Mean of all parameters plotted on a more detailed scale than in A.
C. SD of all parameters as a function of the number of cycles.
D. CV of all parameters as a function of the number of cycles.
E. CV for 1, 3 and 5 circadian cycles plotted together for comparison.

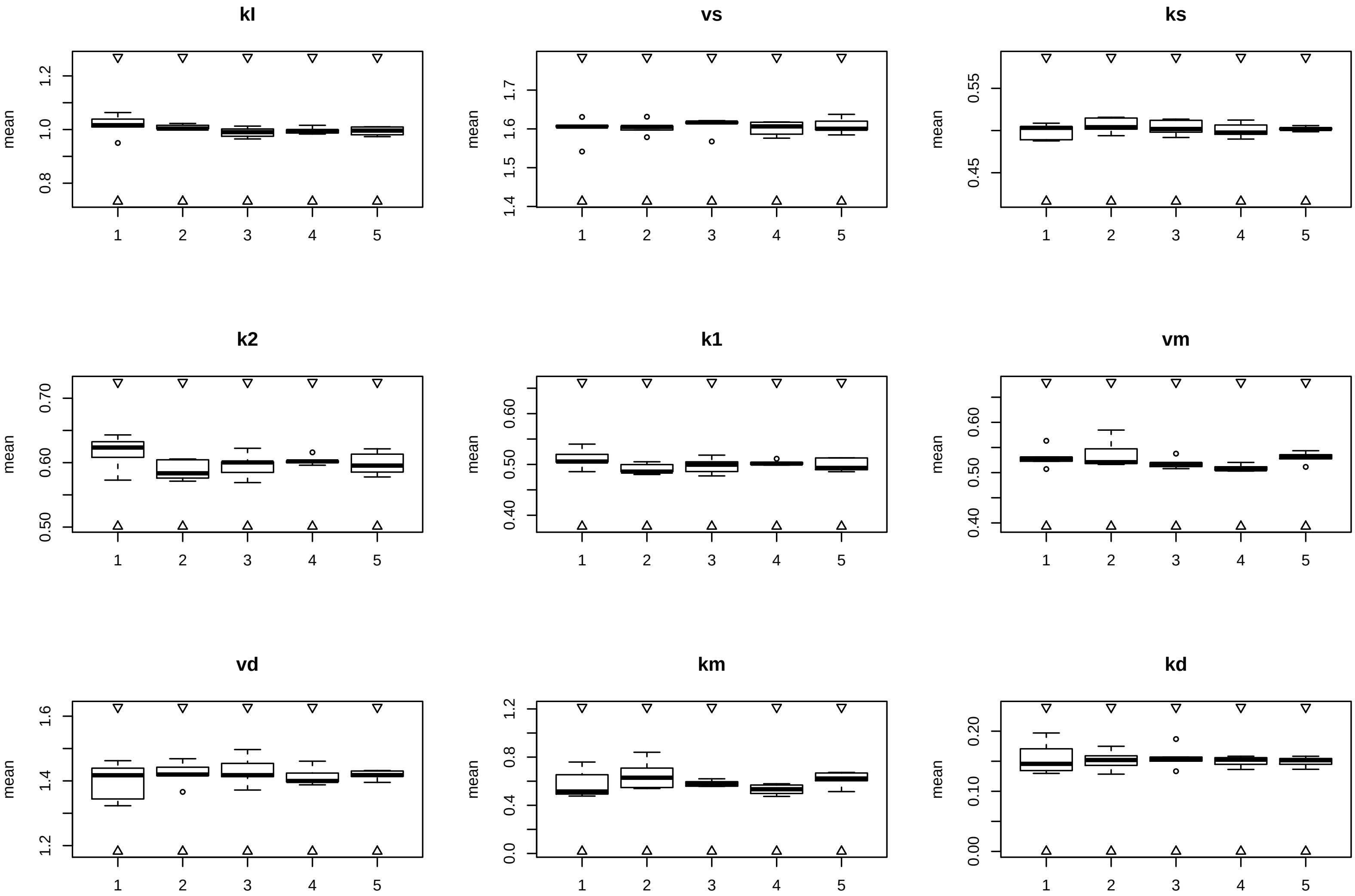

Fig. S7A. Mean of all parameters as a function of the number of cycles. Triangles indicate the prior width.

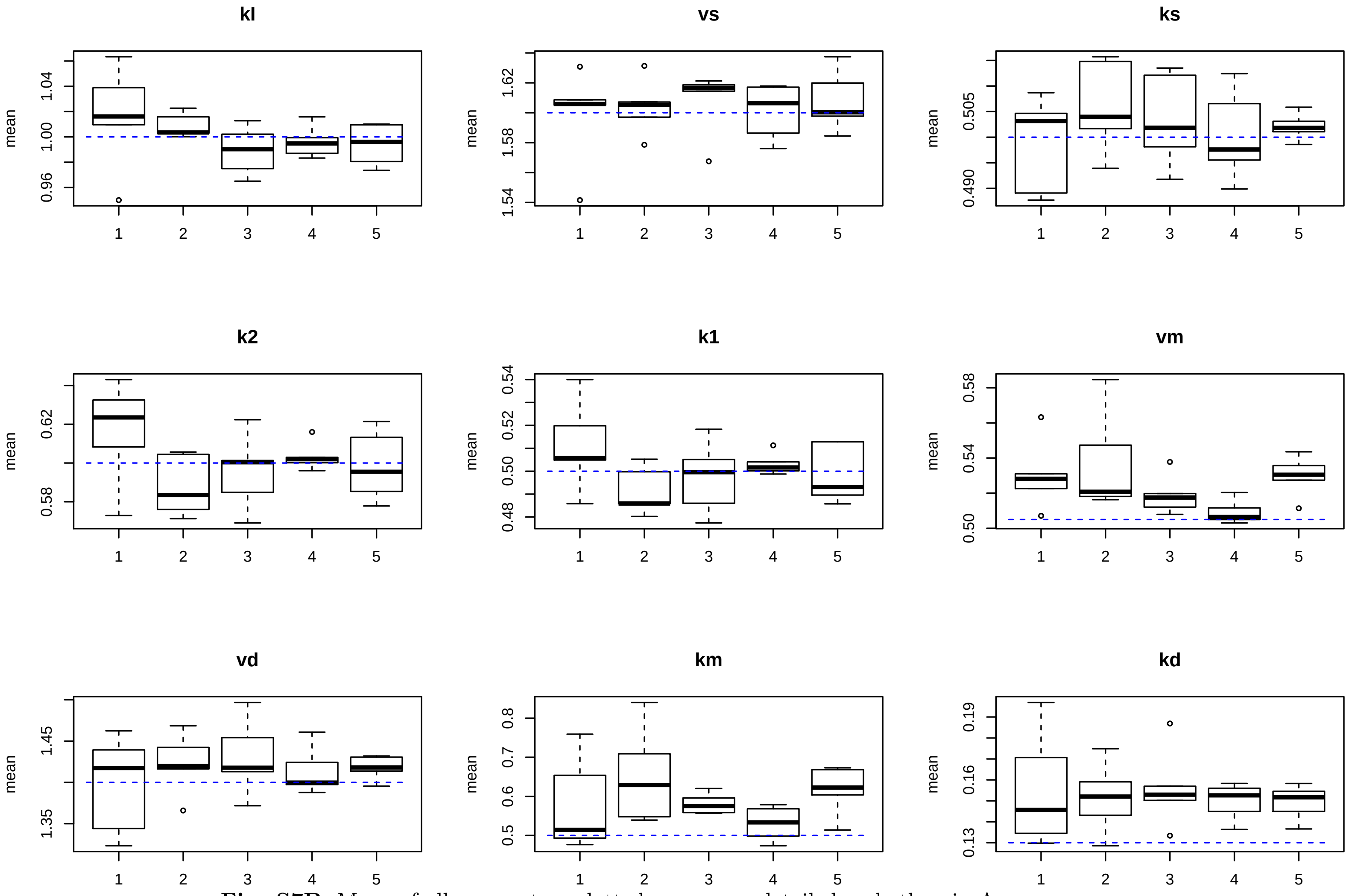

Fig. S7B. Mean of all parameters plotted on a more detailed scale than in A.

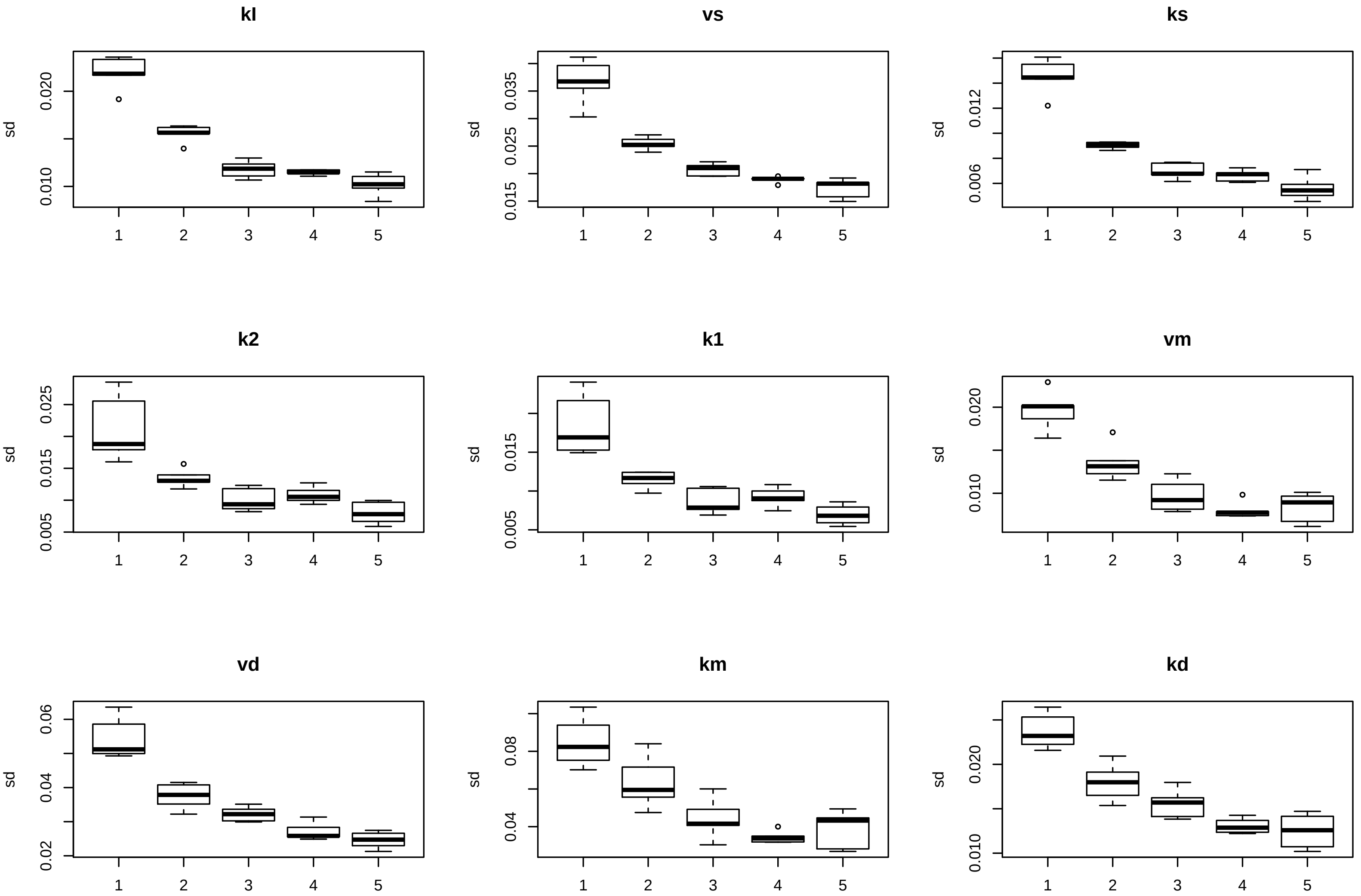

Fig. S7C. SD of all parameters as a function of the number of cycles.

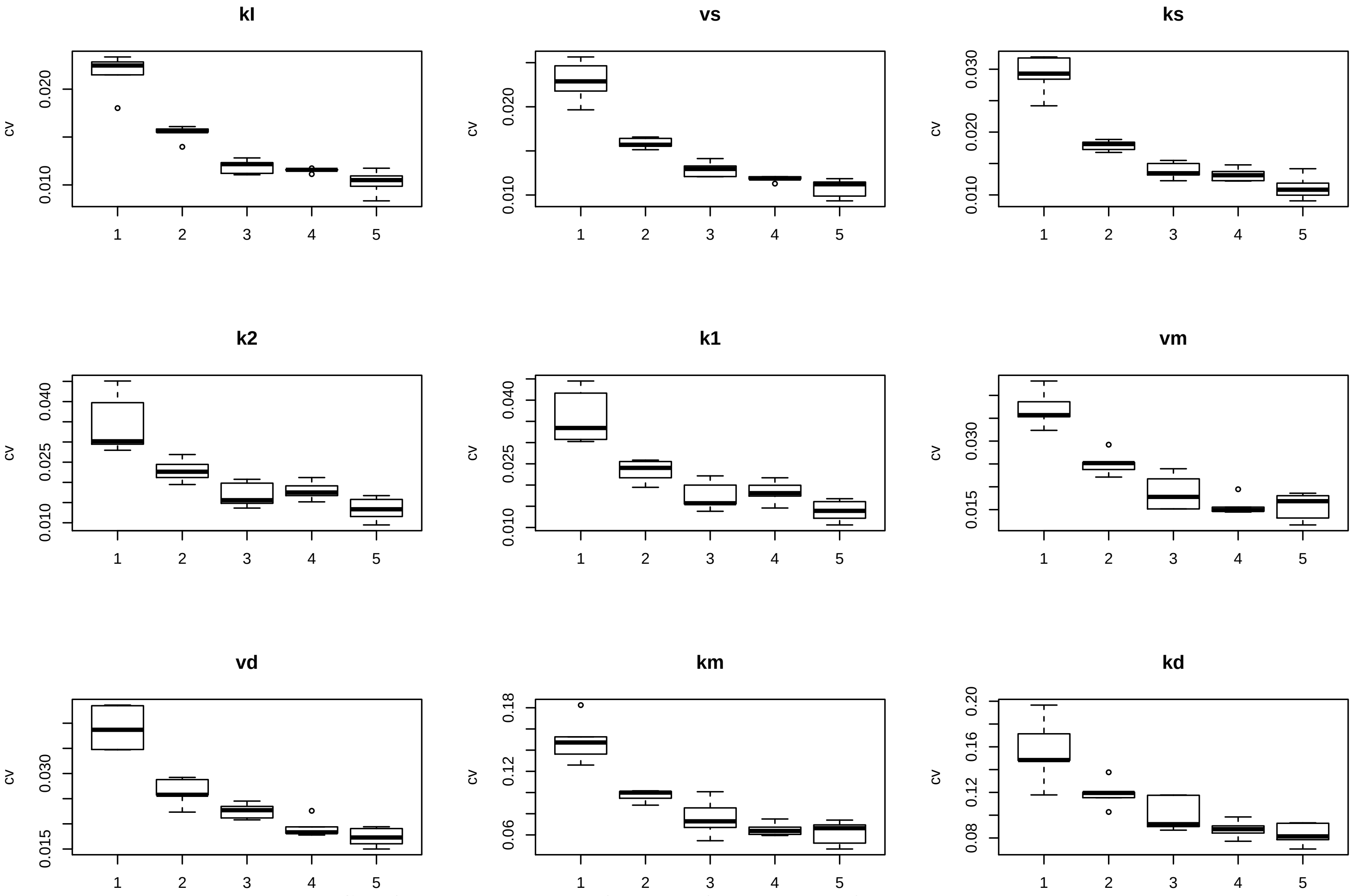

Fig. S7D. CV of all parameters as a function of the number of cycles.

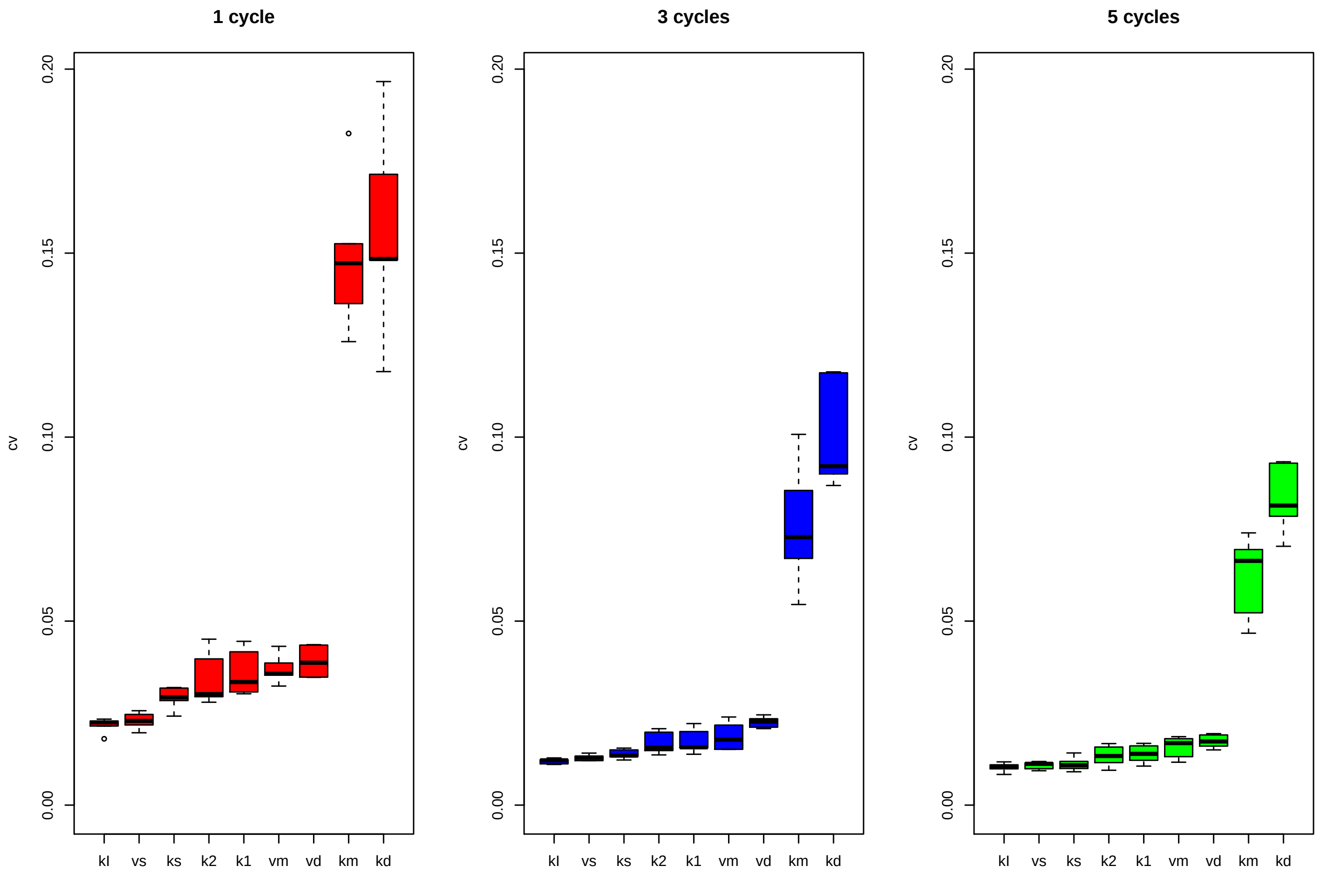

Fig. S7E. CV for 1, 3 and 5 circadian cycles plotted together for comparison.

3 Comparison of nested sampling and MCMC

A single realisation of the DD model was analysed by five repetitions of nested sampling and by a standard implementation of MCMC [1]. (Note that all other analyses apply nested sampling to five different realisations of the circadian model.) In both algorithms, the lower and upper parameter bounds are mapped to 0..1 in all dimensions, and hence inference takes place within a unit cube. The output analysis of MCMC followed the recommendations in [1]. An average of 57,018 log likelihood calls were required to obtain 1054 posterior samples in nested sampling, whereas MCMC required 150,000 evaluations to obtain 1500 samples (using a batch of length 100, acceptance rate 11%). The acceptance ratio of MCMC is strongly influenced by the scale parameter which sets the proposal step size ($scale * \mathcal{N}(0, 1)$). Alternative scales were explored to ensure consistent results from MCMC simulations of the circadian model, and from multi-dimensional Gaussian test problems. One complication that arises in high-dimensional problems is that lower values for scale improve the acceptance ratio but increase the computation required to explore the prior.

All nine model parameters are approximately normally distributed as indicated by the sample histograms (Fig. S8A) and QQ plots (Fig. S8B) of MCMC samples. On the assumption that parameters are normally distributed, a graphical comparison of the (theoretical) normal densities inferred by nested sampling can be made with those inferred by MCMC, see Fig. S8C. The densities plotted for the most typical run of nested sampling (the run giving the greatest number of median estimates of parameter means (4 parameters of 9)). While some differences in the location of the means can be noted, the predicted posterior densities are in generally good agreement.

Fig. S8. Parameter inference by nested sampling and by MCMC. Nested sampling was run five times on the same realisation of one circadian cycle of the the free-running (DD) model and the most typical result selected. A single long run of MCMC provided 1500 posterior samples for comparison.
A. Distributions of all parameters computed by one run of MCMC (the blue circles indicate the parameter value used to generate the data series).
B. QQ plots of the samples in Fig. S8A. indicate that all parameters are approximately normally distributed.
C. Density plots of the normal distribution with mean and standard deviation estimated by MCMC (dashed black lines) and by nested sampling (blue lines).

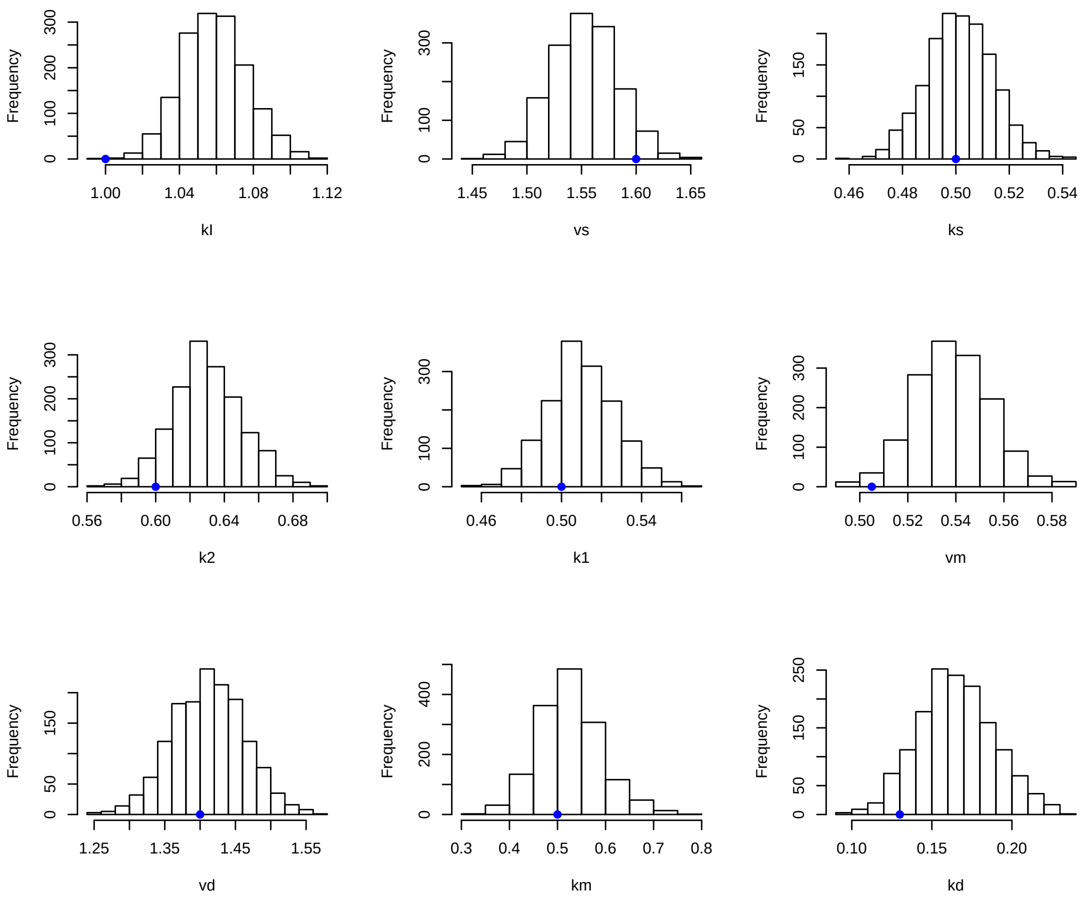

Fig. S8A. Distributions of all parameters computed by one run of MCMC.

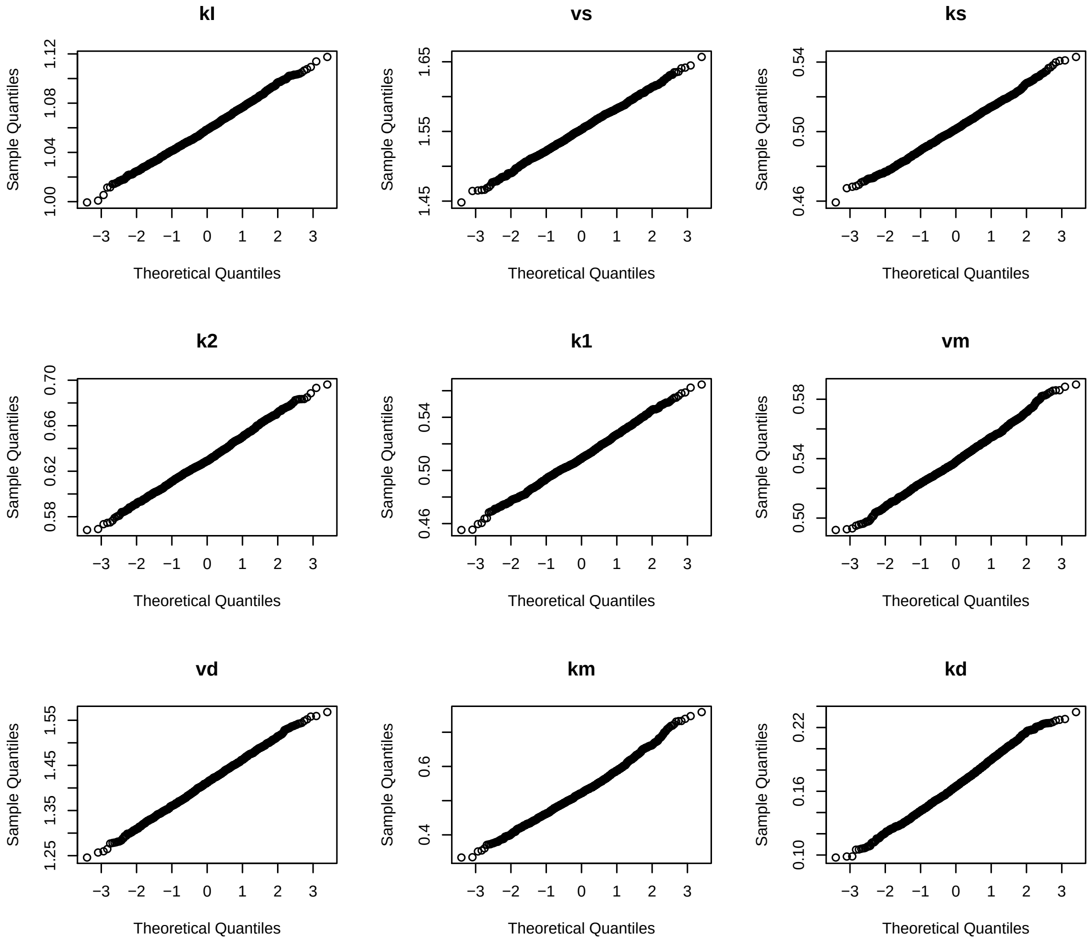

Fig. S8B. QQ plots of the samples in Fig S8A. compared with the normal distribution.

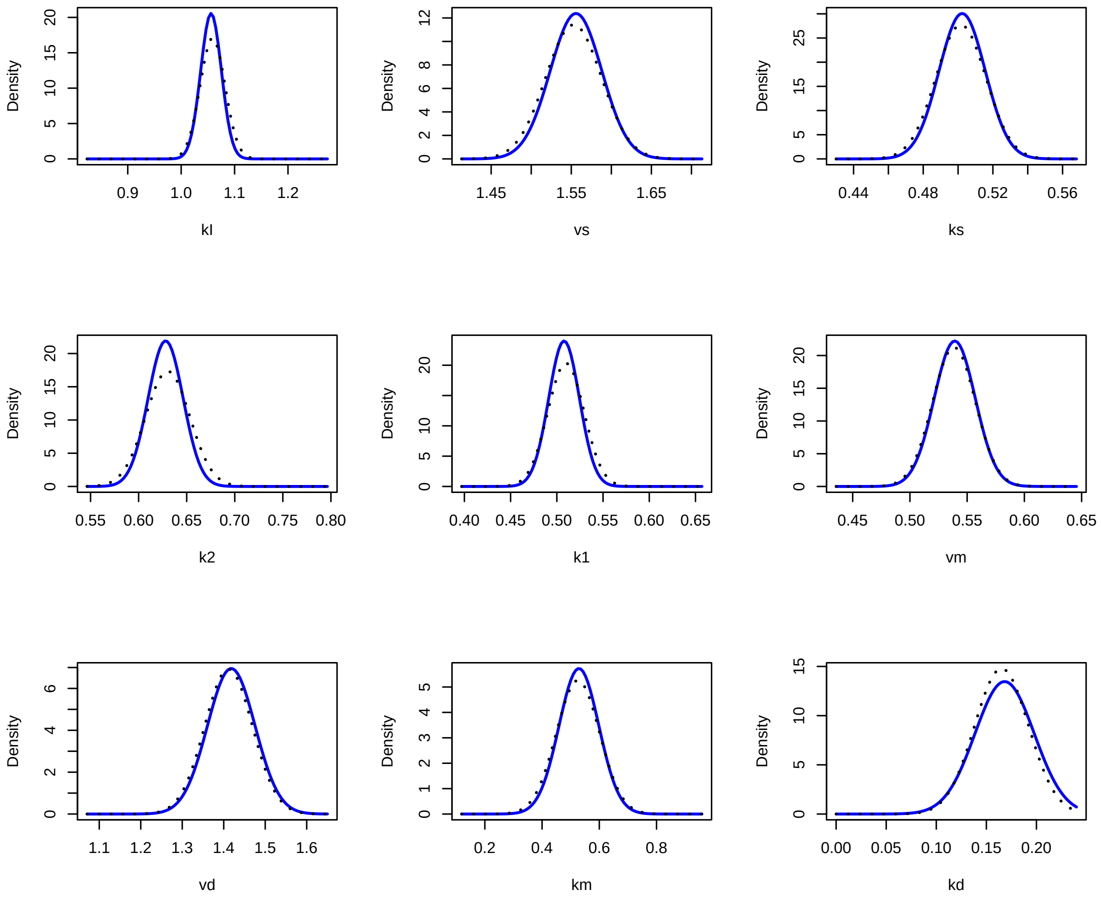

Fig. S8C. Posterior (normal) densities inferred from MCMC (dashed black) and by nested sampling (blue).

4 Parameter inference for the free-running (DD) system with constant sample size

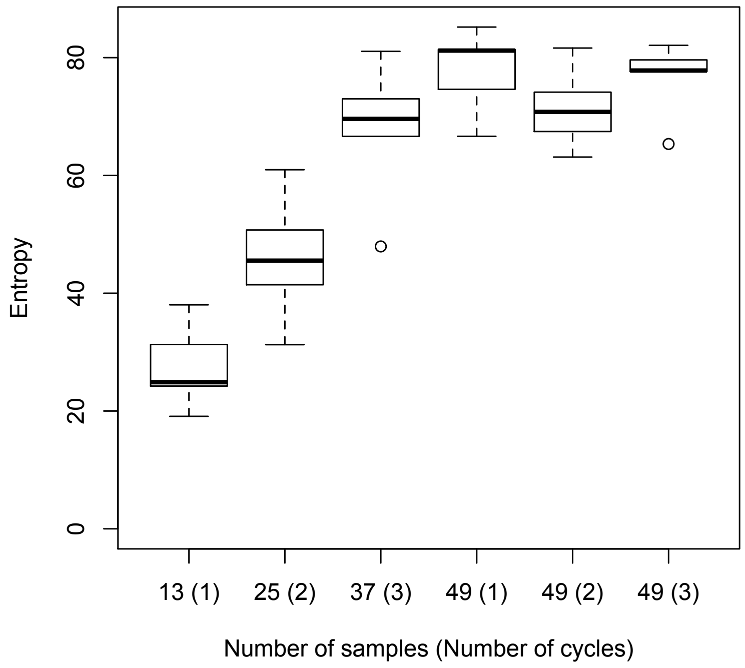

Fig. S9. Entropy of data sampled over 1-3 circadian cycles. Total entropy of 13, 25 and 37 samples, and of 49 samples obtained from 1, 2 and 3 circadian cycles respectively. Total entropy is defined as the sum of $p\ log(p)$ for all bridge points (p is calculated by (equation (18) in the main text)). Entropy increases with increasing numbers of samples, and is approximately equal when 49 samples are selected from 1-3 circadian cycles.

Fig. S10. Parameter inference for the free-running (DD) system over 1-3 circadian cycles of data where 49 samples are selected from each data set. All figures show box plots summarising 5 runs of the nested sampling algorithm. Each run used a different stochastic realisation of the circadian model. The results are grouped and plotted in several different ways to ease comparison. The parameter values used to generate each of the synthetic data series are indicated by the blue dashed lines.

A. Mean of all parameters as a function of the number of cycles. Triangles indicate the upper and lower bound used for integration (prior width).

B. Mean of all parameters plotted on a more detailed scale than in A.

C. SD of all parameters as a function of the number of cycles.

D. CV of all parameters as a function of the number of cycles.

E. CV for 1-3 circadian cycles plotted together for comparison.

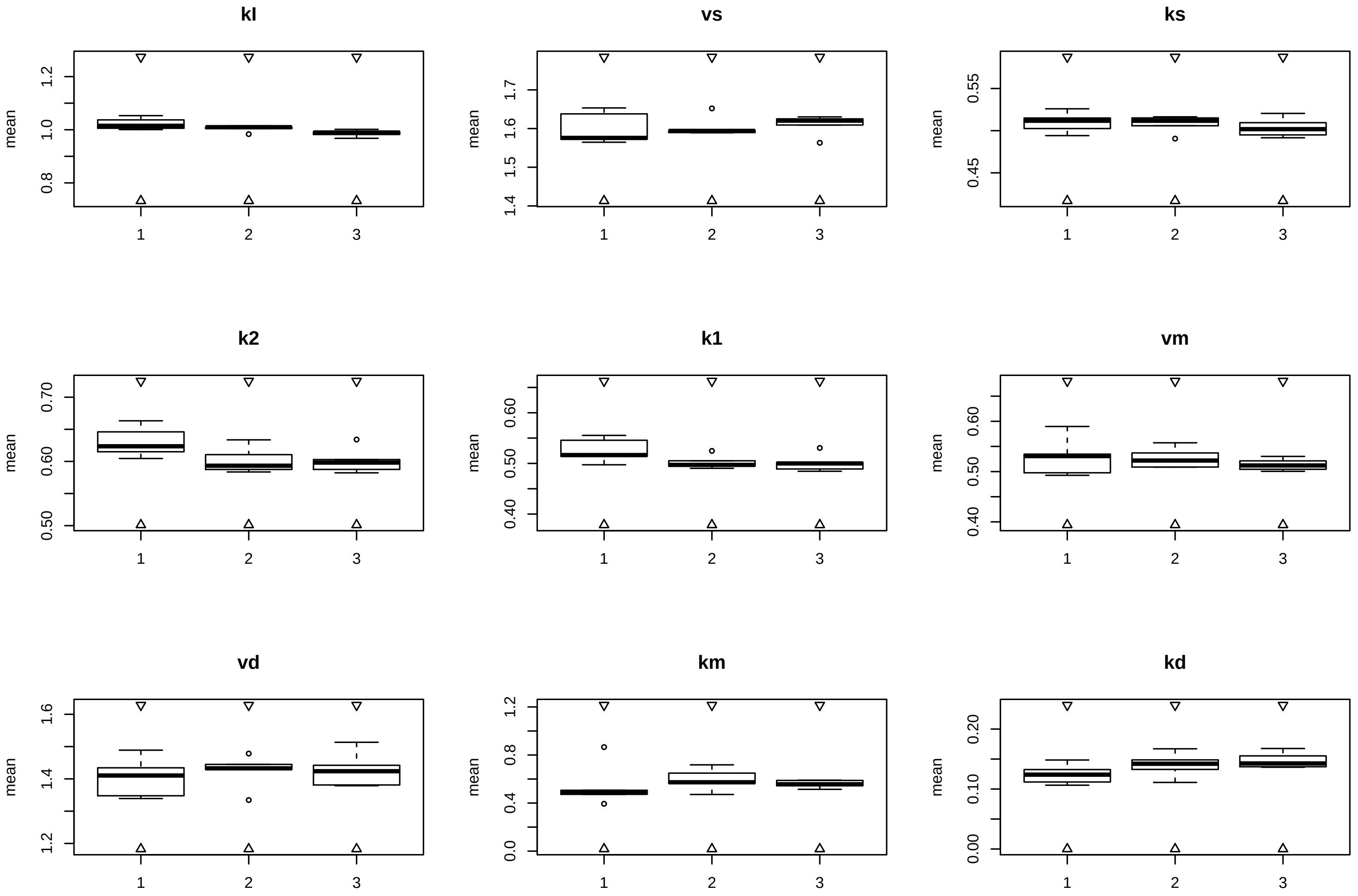

Fig. S10A. Mean of all parameters as a function of the number of cycles. Triangles indicate the prior width.

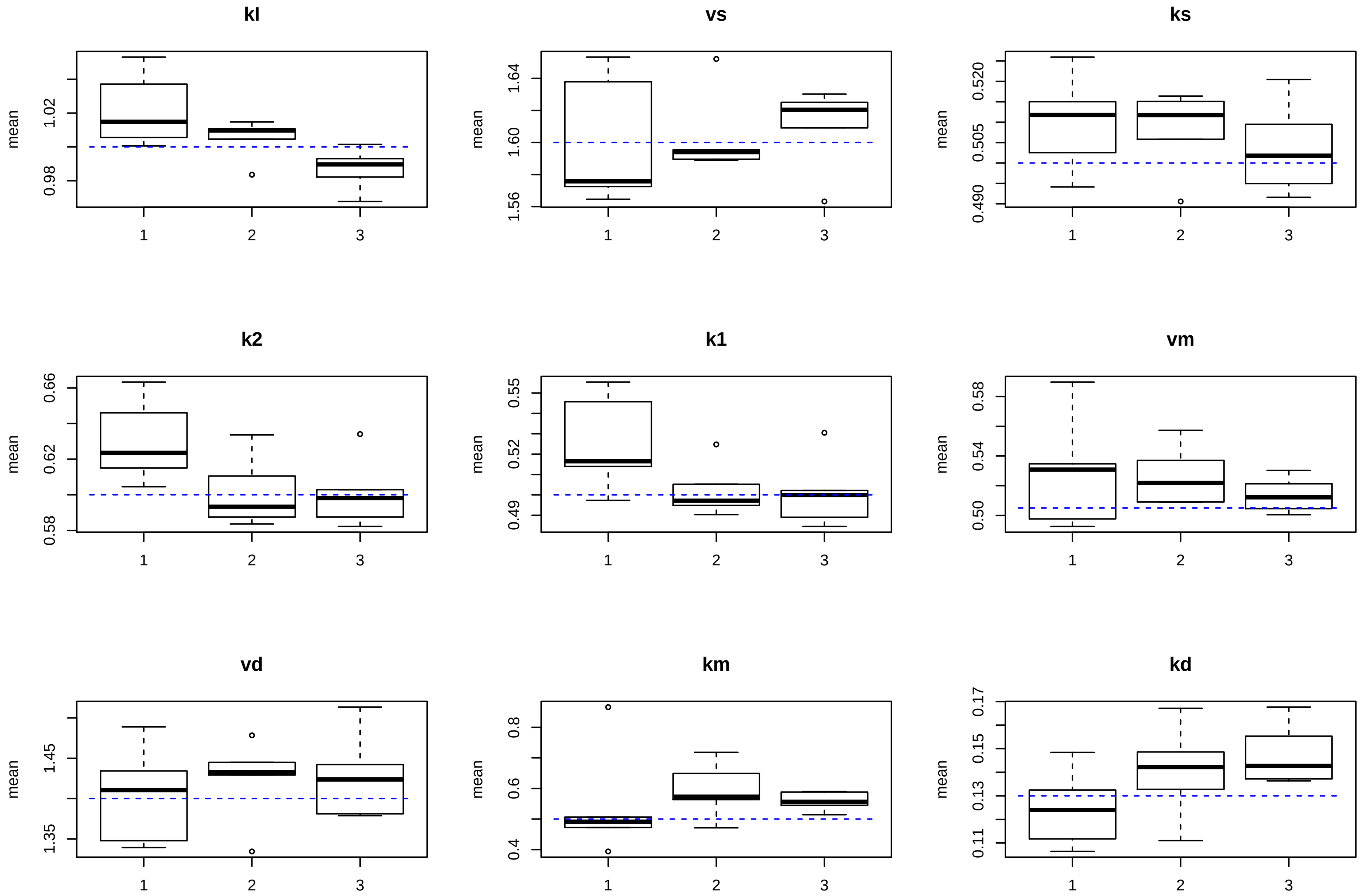

Fig. S10B. Mean of all parameters on a more detailed scale than in A.

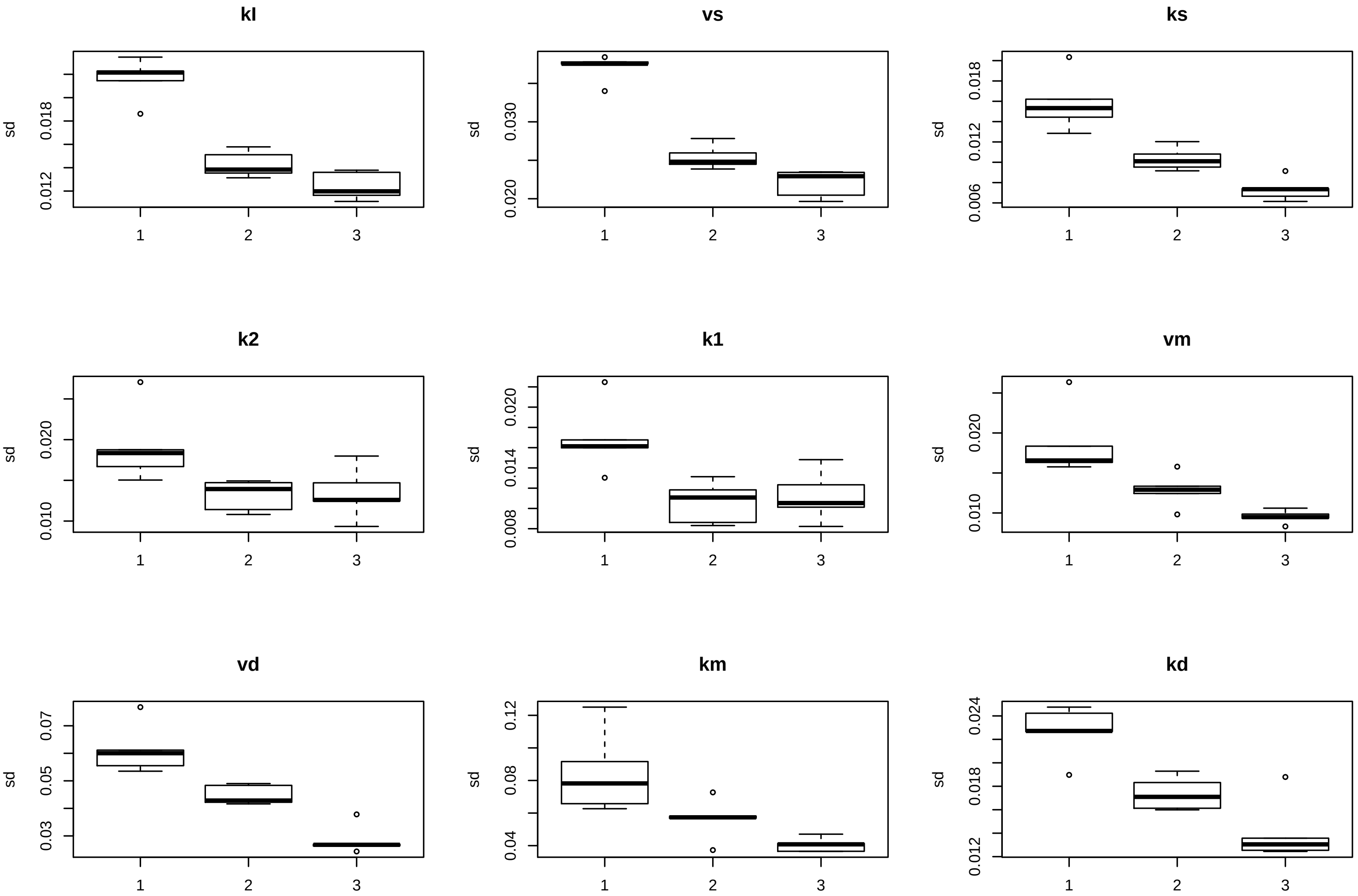

Fig. S10C. SD of all parameters as a function of the number of cycles.

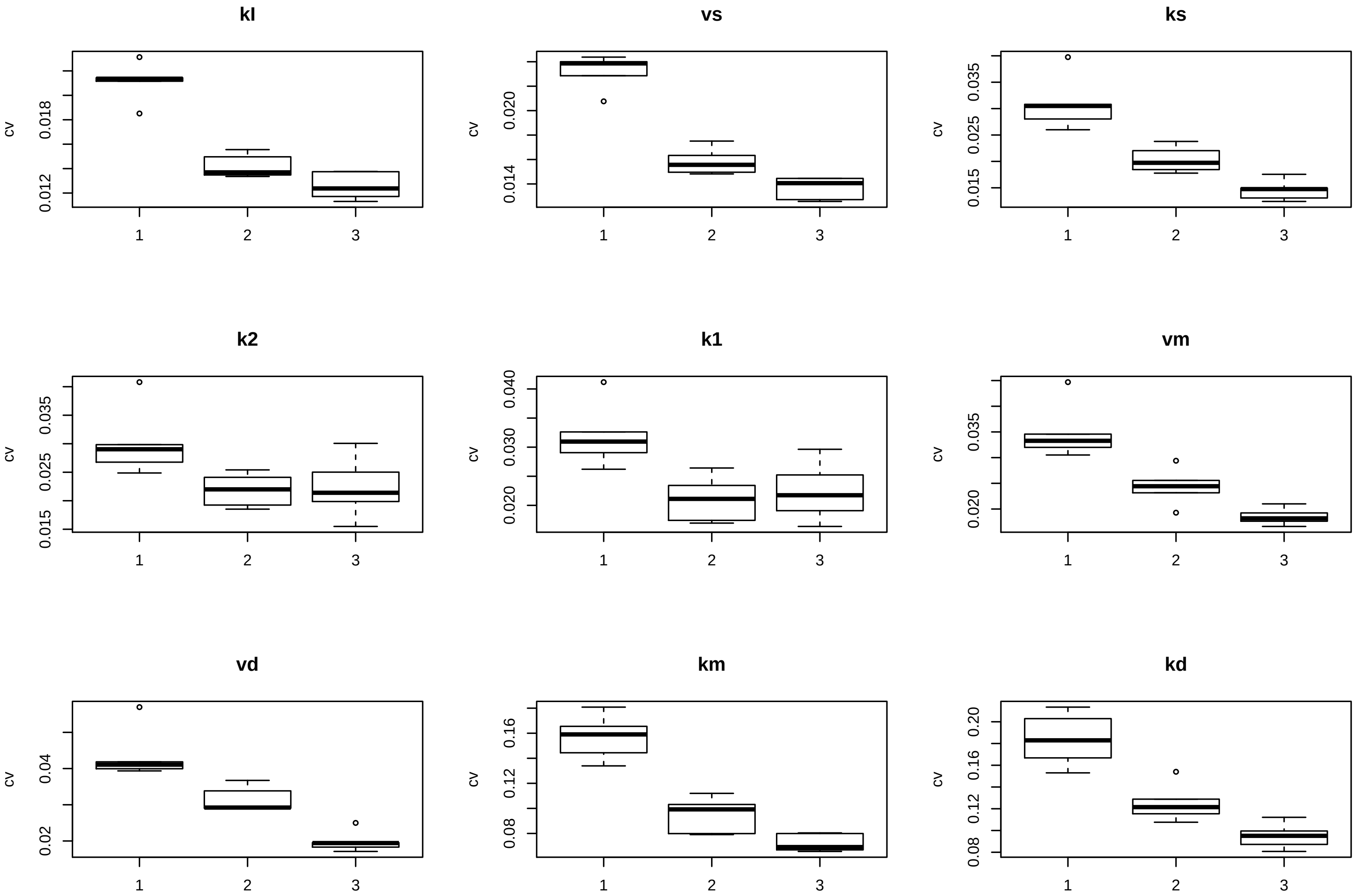

Fig. S10H. CV of all parameters as a function of the number of cycles.

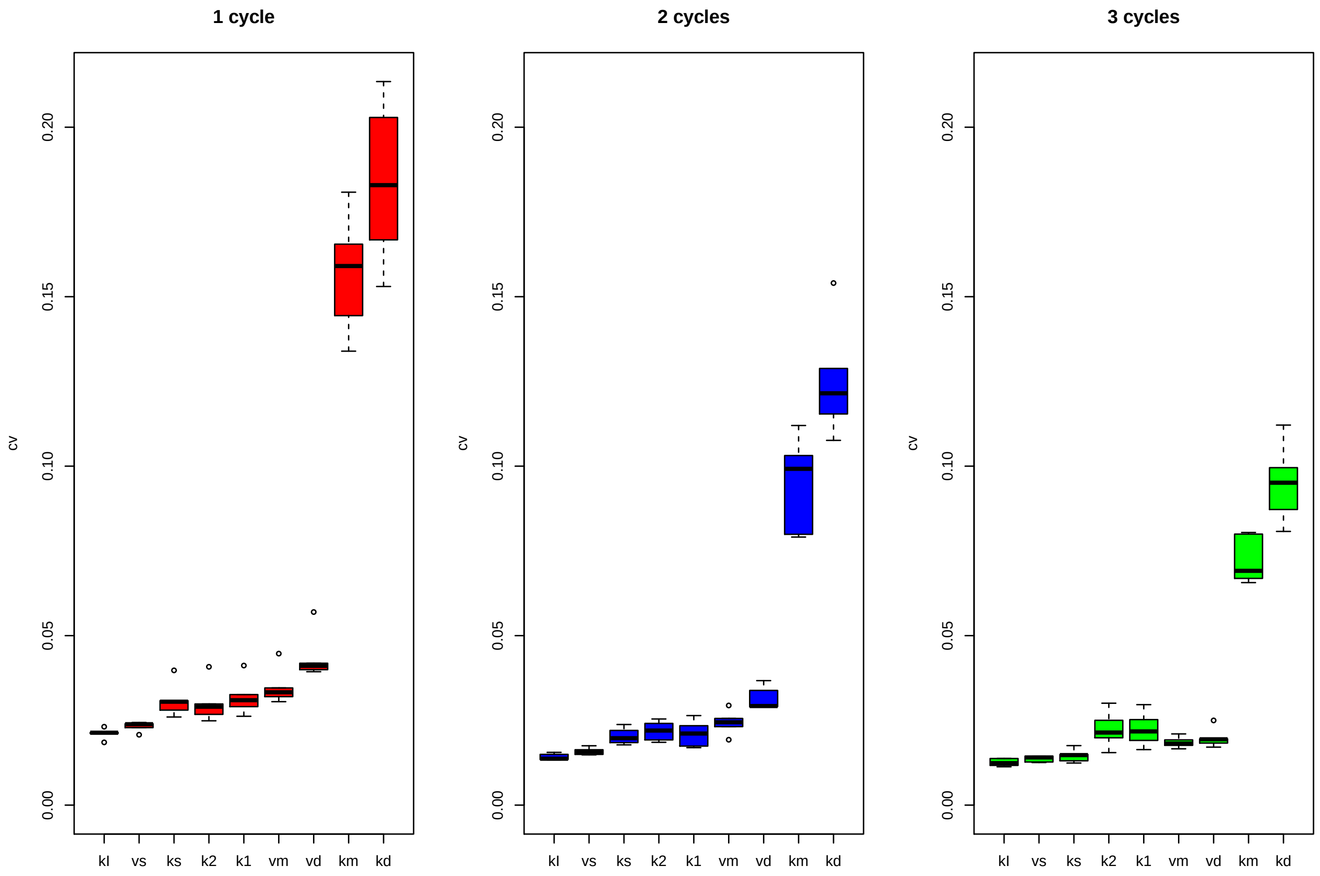

Fig. S10D. CV for 1-3 circadian cycles plotted together for comparison.

5 Parameter inference for LD protocols

Fig. S11. Parameter inference for DD and for three LD protocols for 1, 3 and 5 circadian cycles of data. All figures show box plots summarising 5 runs of the nested sampling algorithm for the free-running system (DD), 8, 12 and 16 hours of light (8L:16D, 12L:12D and 16L:8D respectively). Each run used a different stochastic realisation of the circadian model. The parameter values used to generate each of the synthetic data series are indicated by the blue dashed lines.
A. Mean of all parameters for 1 circadian cycle of data.
B. Mean of all parameters for 3 circadian cycles of data.
C. Mean of all parameters for 5 circadian cycles of data.
D. SD of all parameters for 1 circadian cycle of data.
E. SD of all parameters for 3 circadian cycles of data.
F. SD of all parameters for 5 circadian cycles of data.
G. CV of all parameters for 1 circadian cycle of data.
H. CV of all parameters for 3 circadian cycles of data.
I. CV of all parameters for 5 circadian cycles of data.

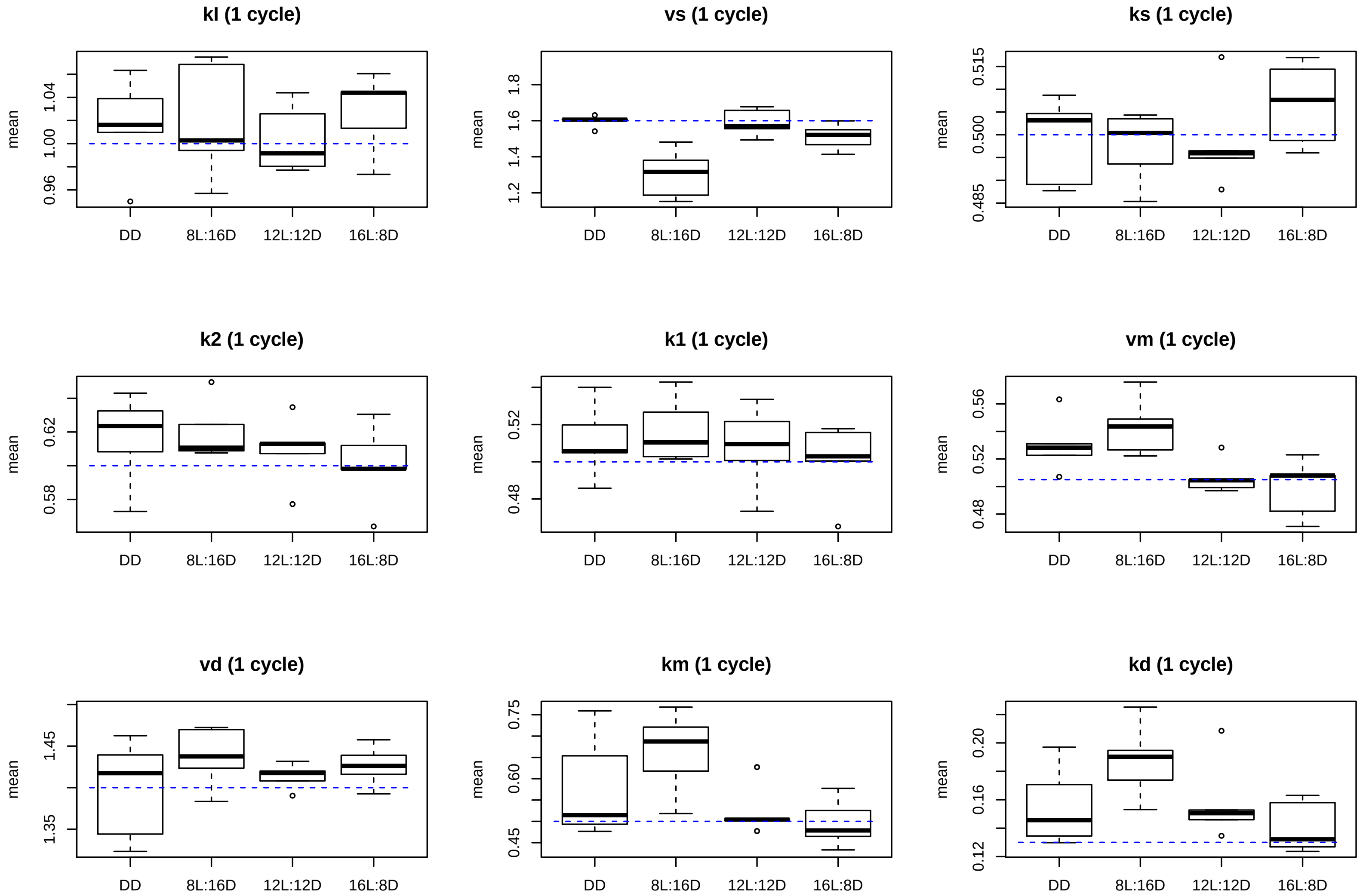

Fig. S11A. Mean of all parameters for 1 circadian cycle of data.

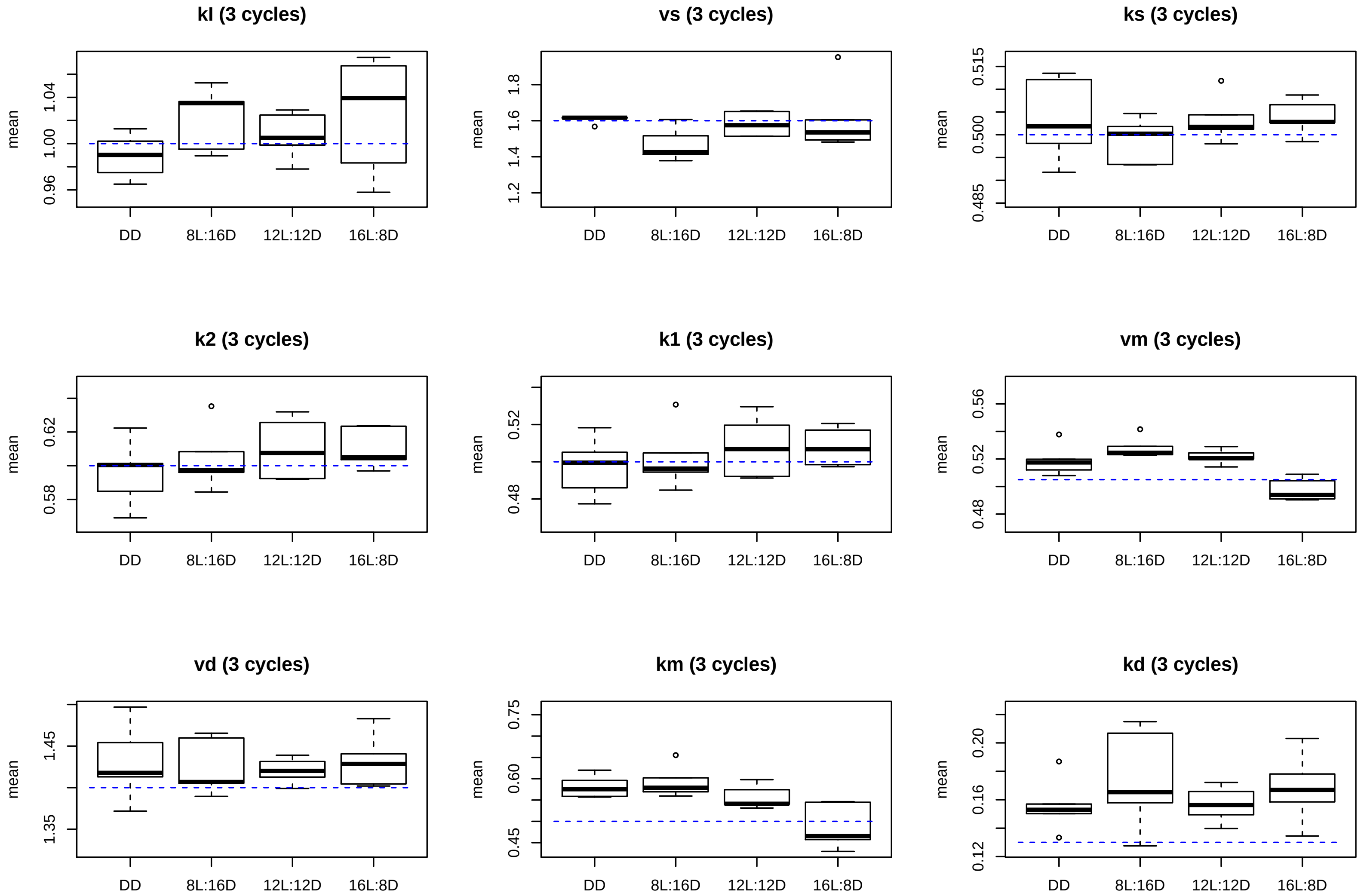

Fig. S11B. Mean of all parameters for 3 circadian cycles of data.

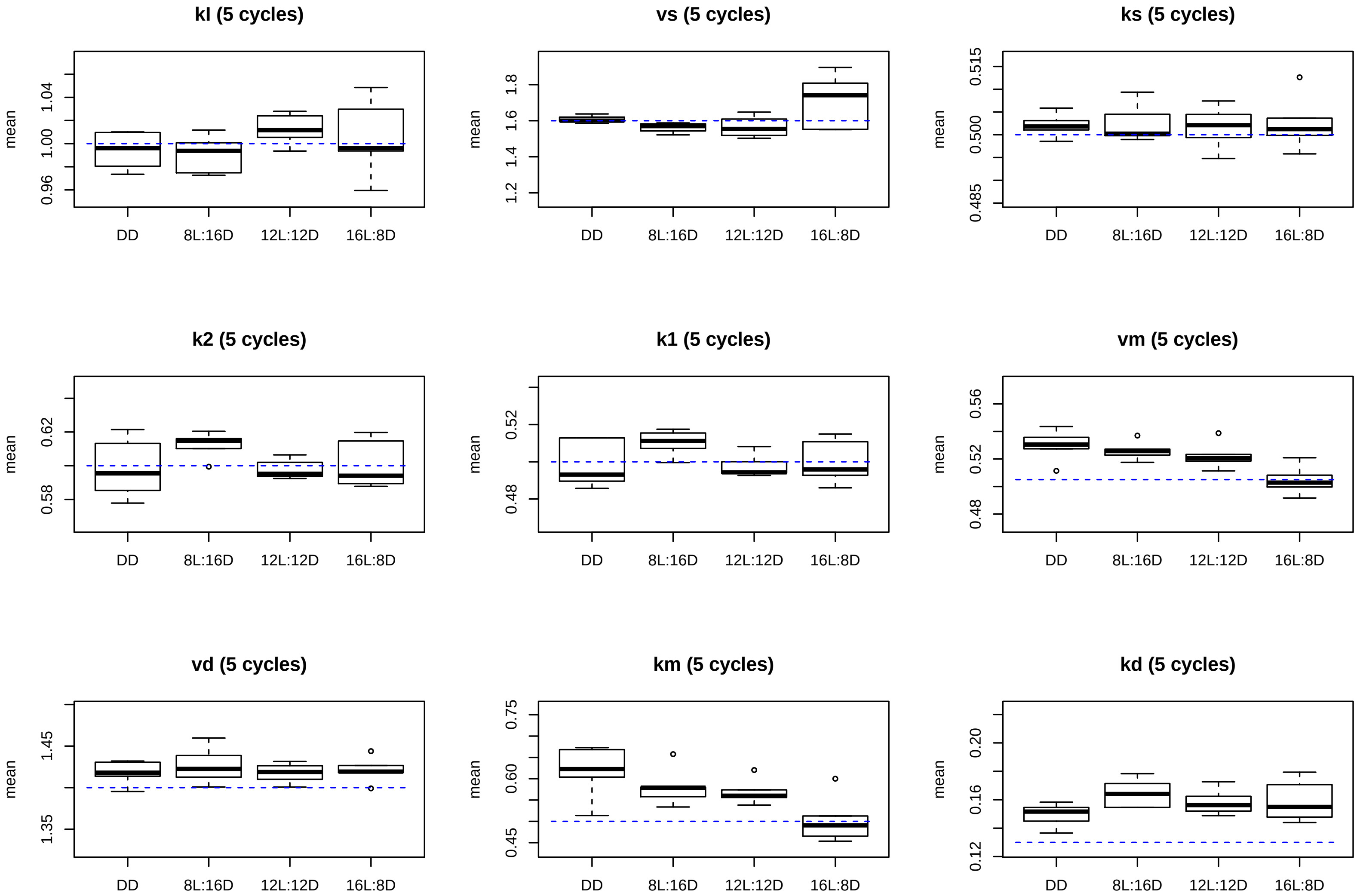

Fig. S11C. Mean of all parameters for 5 circadian cycles of data.

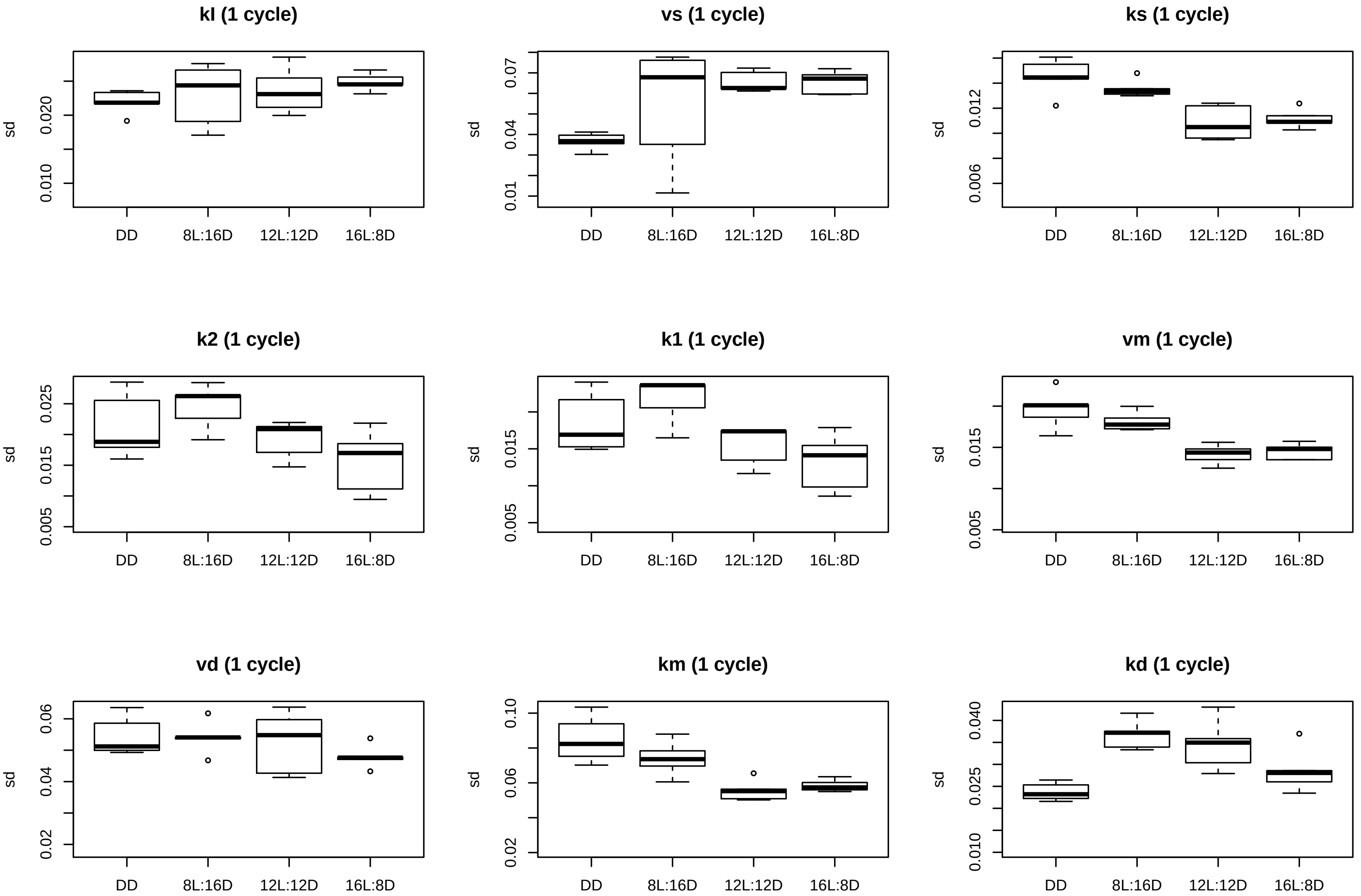

Fig. S11D. SD of all parameters for 1 circadian cycle of data.

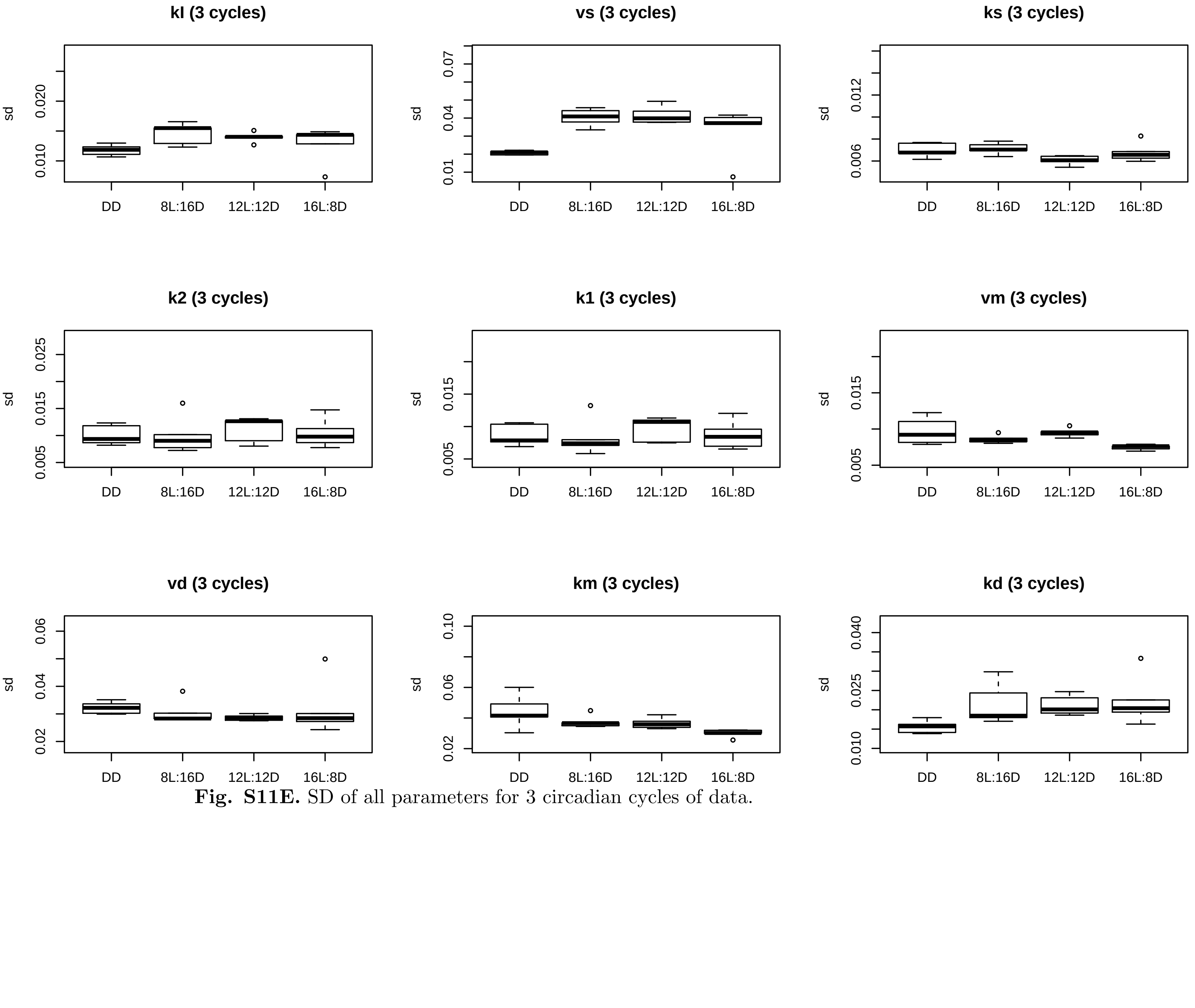

Fig. S11E. SD of all parameters for 3 circadian cycles of data.

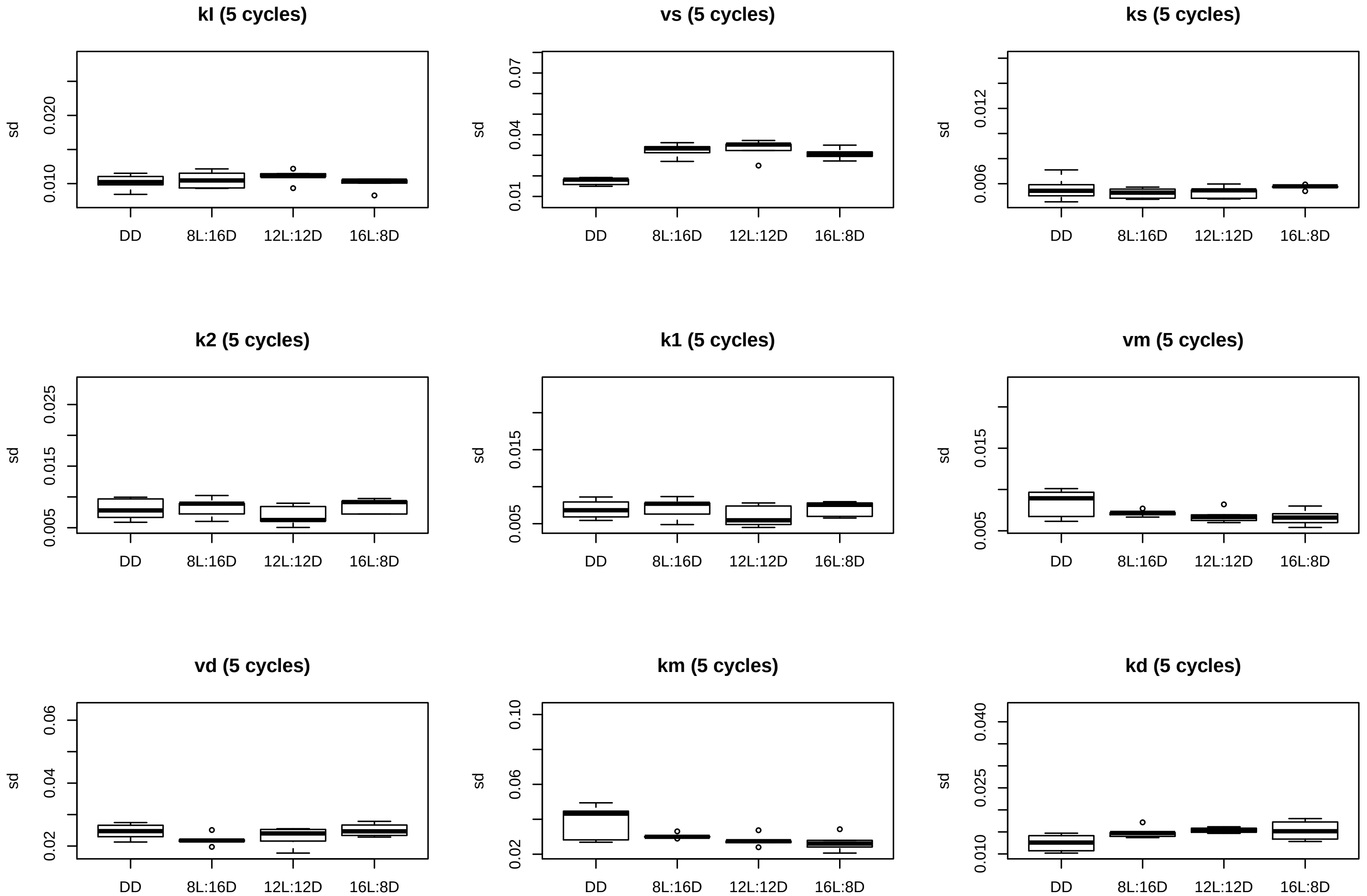

Fig. S11F. SD of all parameters for 5 circadian cycles of data.

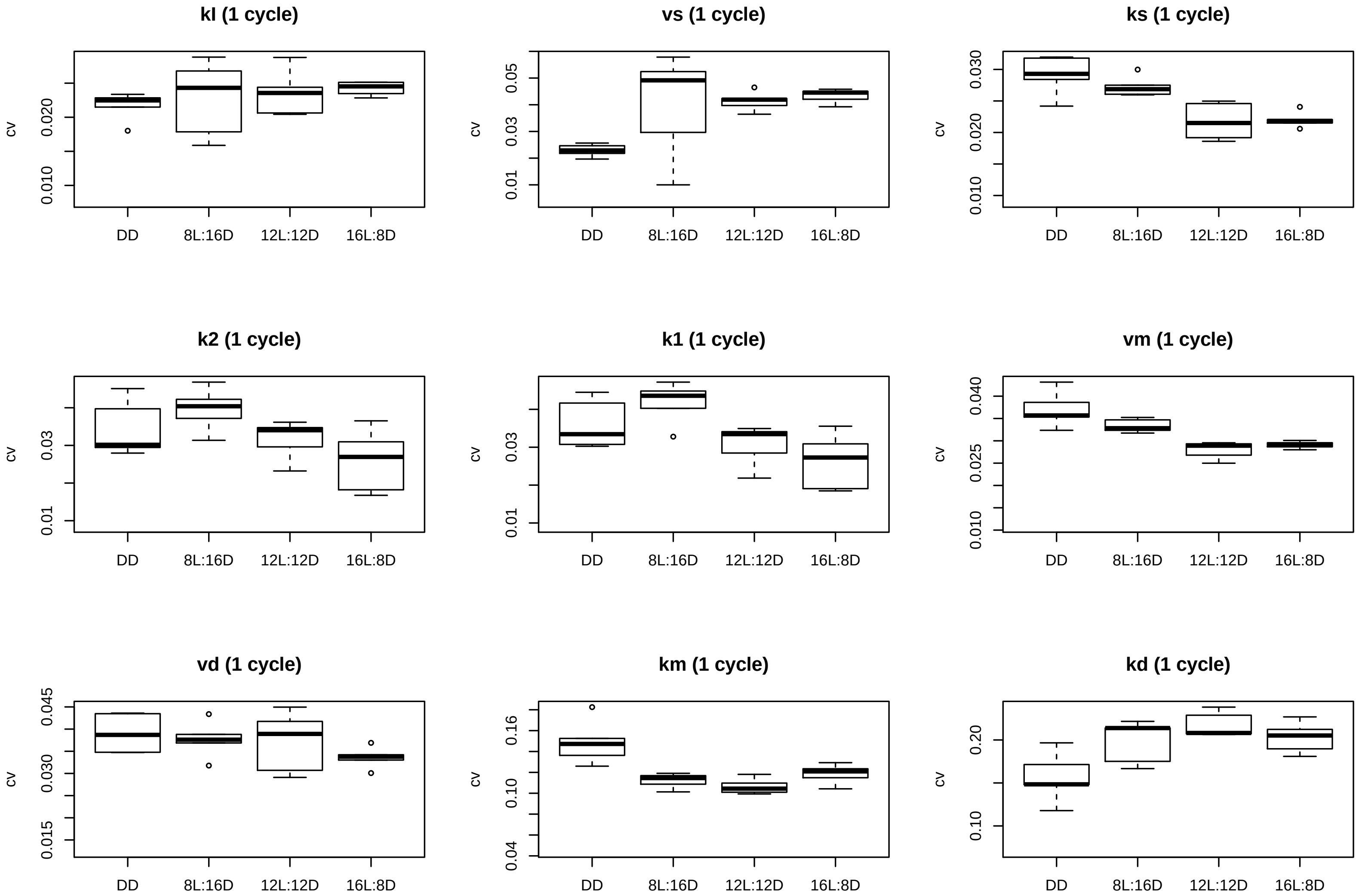

Fig. S11G. CV of all parameters for 1 circadian cycle of data.

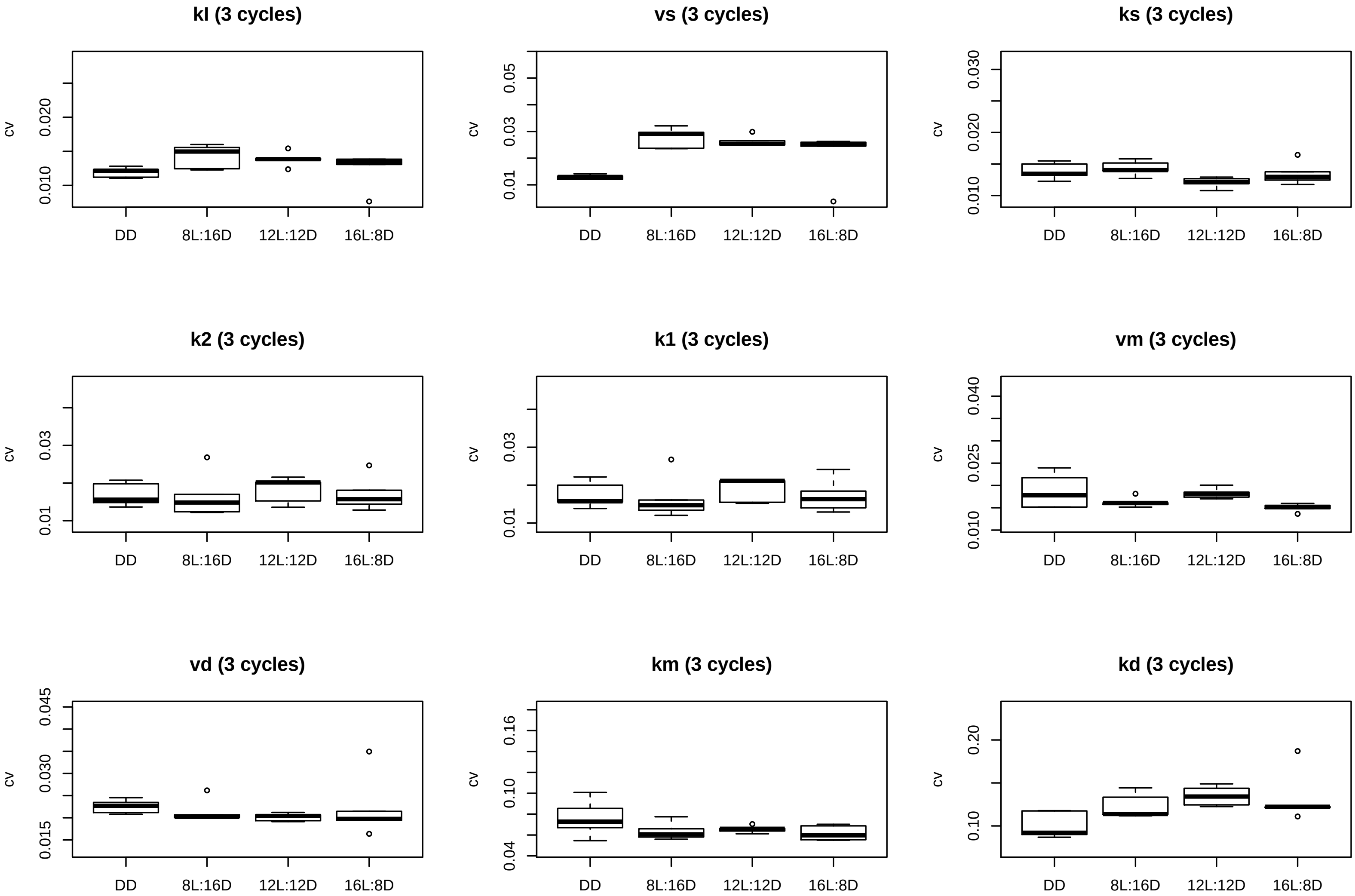

Fig. S11H. CV of all parameters for 3 circadian cycles of data.

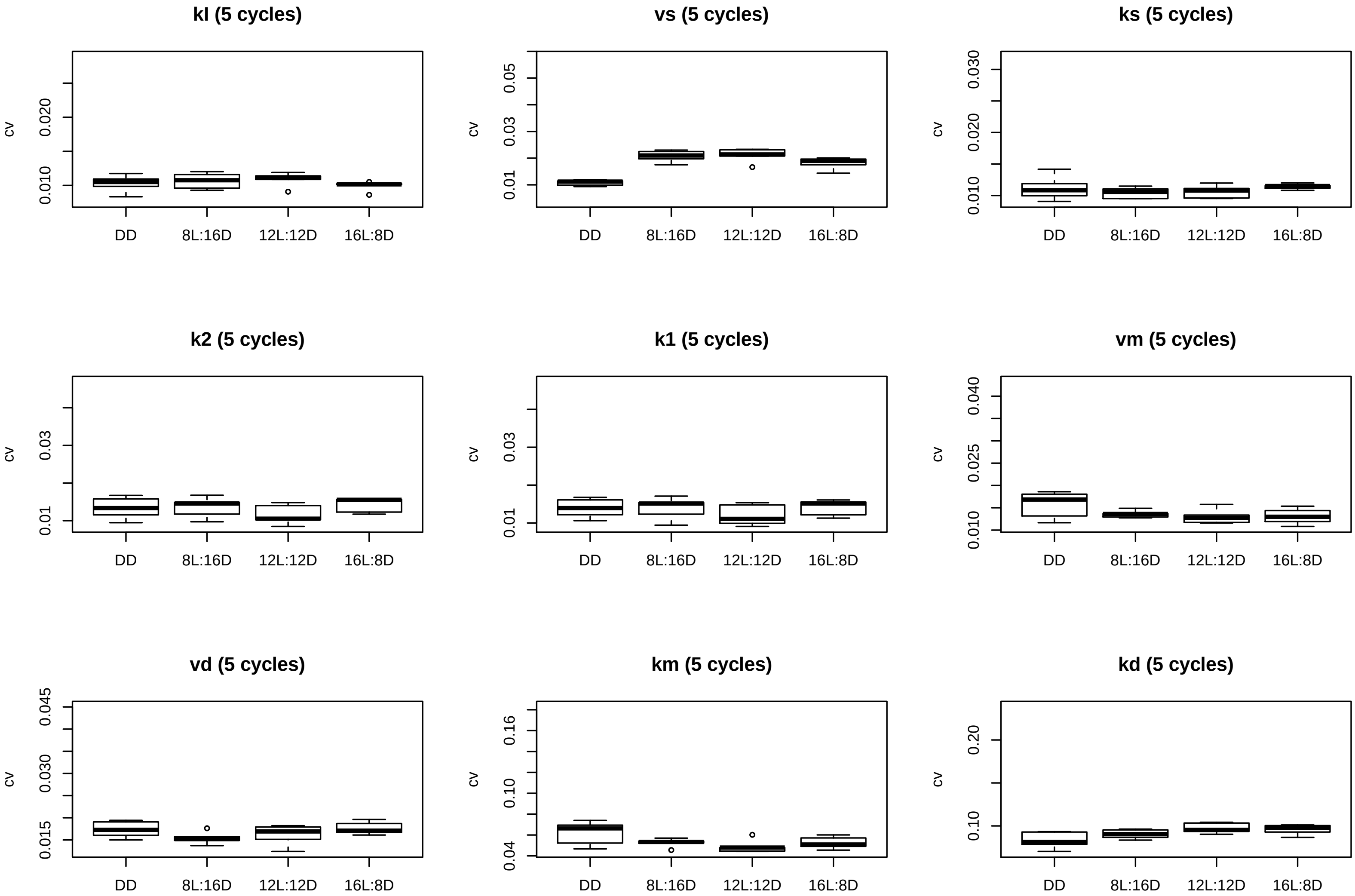

Fig. S11I. CV of all parameters for 5 circadian cycles of data.

6 Free-running Circadian Model

```
// Leloup 1999 - Free-running system (DD)
#model "Leloup_Neurospora_DD";

// rates and constants
vs=1.6;
ks=0.5;
k1=0.5;
k2=0.6;
vm=0.505;
vd=1.4;
kI=1.0;
n=4; //Hill coefficient
km=0.5;
kd=0.13;

omega = 500; //system size

// species
M  = omega*0.2930;
Pc = omega*1.0046;
Pn = omega*1.6590;

// model
frq_transc,  ->  M, [vs*(omega*kI^n)/((kI^n) + ((Pn/omega)^n))];
FRQ_transl,M -> M + Pc, ks;
nuc_transp_f,  Pc -> Pn, k1;
nuc_transp_b,Pn -> Pc, k2;
frq_deg,M -> , [vm*(M/(km+(M/omega)))];
FRQ_deg,Pc -> , [vd*(Pc/(kd+(Pc/omega)))];

/// end
```

7 Entrained Circadian Model

```
// Leloup 1999 - Entrained system (forced by light-dark (LD) cycles)
#model "Leloup_Neurospora_LD";

// rates and constants
vs=1.6; // baseline transcription rate
ks=0.5;
k1=0.5;
k2=0.6;
vm=0.505;
vd=1.4;
kI=1.0;
n=4; //Hill coefficient
km=0.5;
kd=0.13;

amp=0.8; //light parameters
dawn=6;
dusk=18;
cyclen=24.0;

omega = 500; //system size

// species
M  = omega*3.7018;
Pc = omega*6.2216;
Pn = omega*4.6681;

//LD cycle
LDcyc = [amp*theta((time-cyclen*floor(time/cyclen))-dawn)
        *theta(dusk-(time-cyclen*floor(time/cyclen)))];

// model
frq_transc,  -> M, [(vs+LDcyc)*(omega*kI^n)/((kI^n) + ((Pn/omega)^n))];
FRQ_transl,M -> M + Pc, ks;
nuc_transp_f,  Pc -> Pn, k1;
nuc_transp_b,Pn -> Pc, k2;
frq_deg,M -> , [vm*(M/(km+(M/omega)))];
FRQ_deg,Pc -> , [vd*(Pc/(kd+(Pc/omega)))];

/// end
```

References

[1] Geyer C: **Practical Markov Chain Monte Carlo**. *Statistical Science* 1992, **7**(4):473–511.
